# Trans-synaptic and retrograde axonal spread of Lewy pathology following pre-formed fibril injection in an in vivo A53T alpha-synuclein mouse model of synucleinopathy

**DOI:** 10.1186/s40478-020-01026-0

**Published:** 2020-08-28

**Authors:** Allison J. Schaser, Teresa L. Stackhouse, Leah J. Weston, Patrick C. Kerstein, Valerie R. Osterberg, Claudia S. López, Dennis W. Dickson, Kelvin C. Luk, Charles K. Meshul, Randall L. Woltjer, Vivek K. Unni

**Affiliations:** 1 Department of Neurology and Jungers Center for Neurosciences Research, Oregon Health and Science University, Portland, OR 97239 USA; 2 Vollum Institute, Oregon Health and Science University, Portland, OR 97239 USA; 3 Multiscale Microscopy Core, Oregon Health and Science University, Portland, OR 97239 USA; 4 Department of Neuroscience, Mayo Clinic, Jacksonville, FL 32224 USA; 5 Department of Pathology and Laboratory Medicine, Institute on Aging and Center for Neurodegenerative Disease Research, University of Pennsylvania Perelman School of Medicine, Philadelphia, PA 19104 USA; 6 Research Services, Veterans Affairs Medical Center, Portland, OR 97239 USA; 7 Department of Behavioral Neuroscience, Oregon Health and Science University, Portland, OR 97239 USA; 8 Department of Pathology, Oregon Health and Science University, Portland, OR 97239 USA; 9 Parkinson Center, Department of Neurology, Oregon Health and Science University, Portland, OR 97239 USA

**Keywords:** Synucleinopathies, Parkinson’s disease, Dementia with Lewy bodies, Alpha-synuclein, Lewy body, Trans-synaptic spread, Neurodegeneration

## Abstract

It is necessary to develop an understanding of the specific mechanisms involved in alpha-synuclein aggregation and propagation to develop disease modifying therapies for age-related synucleinopathies, including Parkinson’s disease and Dementia with Lewy Bodies. To adequately address this question, we developed a new transgenic mouse model of synucleinopathy that expresses human A53T SynGFP under control of the mouse prion protein promoter. Our characterization of this mouse line demonstrates that it exhibits several distinct advantages over other, currently available, mouse models. This new model allows rigorous study of the initial location of Lewy pathology formation and propagation in the living brain, and strongly suggests that aggregation begins in axonal structures with retrograde propagation to the cell body. This model also shows expeditious development of alpha-synuclein pathology following induction with small, in vitro-generated alpha-synuclein pre-formed fibrils (PFFs), as well as accelerated cell death of inclusion-bearing cells. Using this model, we found that aggregated alpha-synuclein somatic inclusions developed first in neurons, but later showed a second wave of inclusion formation in astrocytes. Interestingly, astrocytes appear to survive much longer after inclusion formation than their neuronal counterparts. This model also allowed careful study of peripheral-to-central spread of Lewy pathology after PFF injection into the hind limb musculature. Our results clearly show evidence of progressive, retrograde trans-synaptic spread of Lewy pathology through known neuroanatomically connected pathways in the motor system. As such, we have developed a promising tool to understand the biology of neurodegeneration associated with alpha-synuclein aggregation and to discover new treatments capable of altering the neurodegenerative disease course of synucleinopathies.

**Electronic supplementary material:**

The online version of this article (10.1186/s40478-020-01026-0) contains supplementary material, which is available to authorized users.

## Introduction

Age-related synucleinopathies including Parkinson disease (PD), Dementia with Lewy bodies (DLB), and Multiple Systems Atrophy (MSA) are common and debilitating neurodegenerative disorders that include both motor and non-motor symptoms [24, 58]. Despite their prevalence and impact on quality of life, there are currently no mechanism-based treatments that slow or halt the disease process involved in these disorders [Reviewed in 27].

Alpha-synuclein is a 140 amino acid protein localized to presynaptic terminals and other cellular structures that is thought to be normally intrinsically disordered and soluble [36], or potentially in a tetrameric conformation [6]. However in synucleinopathies, alpha-synuclein aggregates and forms insoluble intracellular inclusions, the hallmark lesions of this set of diseases [Reviewed in 23]. In neurons, aggregation results in somatic and neuritic inclusions, collectively known as Lewy pathology, but inclusions can also form in non-neuronal cells types, such as glial astrocytic inclusions [70] or Papp-Lantos bodies in oligodendrocytes [56, 74, 75].

A large and growing body of work suggests that one possible mechanism of pathological spread in synucleinopathies is cell-to-cell transfer of aggregated alpha-synuclein [5, 16, 18, 20, 26, 30, 47]. This is referred to as the “prion-like” propagation hypothesis [3, 4, 13, 21] and dovetails with work done on human autopsy tissue by Braak and colleagues, which also suggests a pattern of pathological spread, specifically through neuroanatomically connected pathways [10, 28]. In support of the “prion-like” propagation hypothesis, work in model systems has shown that aggregated alpha-synuclein propagation can be induced by the exogenous application of small, in vitro-generated alpha-synuclein pre-formed fibrils (PFFs). Previous research in vivo has also shown that the application of PFFs causes aggregation of endogenous alpha-synuclein and resultant Lewy pathology, first at the site of injection and then in connected brain regions in a time-dependent manner [29, 34, 47, 54, 63]. This has been suggested to occur via several different possible transport mechanisms, including uptake and transport by circulating microglia [37, 64, 69], movement in the extracellular space [15, 39, 55], and neuronal uptake and intracellular transport [1, 17, 18].

To date, direct evidence for any of these mechanisms in vivo is limited [35, 80], primarily due to the previous inability to track the movement of aggregated alpha-synuclein following PFF injection. In support of the neuronal uptake and transport model, data from cell culture experiments show that both anterograde and retrograde fibril movement within cells is possible [12, 20, 84] and that microtubule-associated axonal transport may be involved [31]. Human pathology studies suggest spread in the retrograde direction, with a pattern of pathology that appears specific to retrograde movement from distal axonal processes to the cell body [79, 80]. However, to our knowledge, direct evidence for the mechanism and direction of axonal transport of aggregated alpha-synuclein in vivo has not been shown. Therefore, the purpose of this study was to test the hypothesis that aggregated alpha-synuclein is propagated from cell-to-cell, and to determine whether this occurs using an anterograde or retrograde trans-synaptic mechanism in vivo.

Utilizing in vivo multiphoton imaging and a new A53T alpha-synuclein transgenic mouse model with accelerated PFF Lewy-like pathology formation, our results show direct in vivo evidence for retrograde spread of aggregated alpha-synuclein pathology. Following PFF injection into the mouse cortex, local neuronal aggregation begins exclusively in axons and then propagates retrogradely, first to somatic and then dendritic structures. Our results demonstrate a time-dependent sequence of pathology where alpha-synuclein inclusions form first in neurons and later primarily in astrocytes. In addition, astrocytes with inclusions appear to survive much longer than neuronal cells with inclusions after initial inclusion formation. Additionally, we built upon previous work by Sacino et al. [63] and performed intramuscular (IM) injections of PFFs into the hind limb musculature of our A53T alpha-synuclein transgenic mouse model and tracked pathology spread over time. We used the periphery as the site of injection to test the model that alpha-synuclein aggregation outside of the central nervous system (CNS) can trigger pathological changes that lead to subsequent aggregation and spread into the CNS, as has been shown to occur in several systems [7, 31, 34, 49, 63, 82]. Our results, combined with previous work, show that PFF IM injection results in the formation of Lewy pathology in the spinal cord and brainstem [63] and then progresses from caudal to more rostral parts of the motor system, eventually including the primary motor cortex. This pattern is strongly suggestive of retrograde trans-synaptic spread through neuroanatomically connected pathways. Using correlated light and electron microscopy (CLEM) techniques, we also show that PFF-induced inclusions in mouse brain are made of fibrillar alpha-synuclein, appropriately modeling Lewy pathology characterized by electron microscopy (EM) in human synucleinopathies [Reviewed in 22] [68, 88]. This new model of synucleinopathy has several distinct technical advantages over other systems, including the ability to clearly identify Lewy pathology based on fluorescence imaging of the tagged enhanced green fluorescent protein (EGFP), without the need for antibody-based histochemical techniques, and a very rapid development of Lewy pathology in vivo within days after PFF injection into the cortex. These characteristics make it a useful tool for understanding the biology of neurodegeneration associated with alpha-synuclein aggregation.

## Materials and methods

### Animals

Animals were housed by OHSU’s Department of Comparative Medicine in a light–dark cycle, temperature and humidity-controlled vivarium, and maintained under ad libitum food and water diet. All experiments were approved by the OHSU IACUC. All experiments were performed in accordance with the relevant guidelines and regulations and every effort was made to minimize the number of animals used and their suffering.

### Mouse model generation

Utilizing the OHSU Transgenic Mouse Model Core, we created a mouse expressing human alpha-synuclein fused to EGFP (C-terminal tag) containing a point mutation at Alanine 53 (GCA > ACA) causing a threonine amino acid change (A53T SynGFP). The A53T SynGFP sequence was cloned into the MoPrp.Xho vector (gift of David Borchelt) at the XhoI site [8] with the following linker sequence between alpha-synuclein and EGFP: GlyThrAlaGlyProGlySerIleAlaThr, as previously described [65]. Expression of the transgene is under transcriptional control of the mouse prion protein promoter. The A53T SynGFP mouse is listed as a key resource in Additional file 1: Table S1.

### Humans tissue

A53T human brain autopsy samples were provided by the Mayo Clinic brain bank in Jacksonville, FL. Control human subject tissue came from de-identified patients with synucleinopathies without known A53T mutations that were seen in the Oregon Alzheimer’s Disease Center (ADC). Brain autopsy from control cases was performed in the ADC neuropathology core. Tissue use was approved by the IRB at OHSU.

### Cranial window surgery

*Port*-*injection animals* A total of 6 (4 Female, 2 Male) A53T SynGFP mice underwent cranial window surgery between the ages of 4 to 12 months old (mean age 5.8 months). Cranial window surgeries were completed with a minor modification to previously published protocols [54]. The surgical procedure was carried out in the exact same manner as previously published, however, the glass coverslip used to create the cranial window was fitted with a pre-drilled hole (port injection) to allow for PFF injection and Lewy pathology induction at a later date. *Long*-*term*-*injection animals* A separate group of 3 (1 Female, 2 Male) A53T SynGFP mice underwent PFF injection (described below) between the ages of 9 and 16 months old (mean age 12.7 months). At 2 months post-injection, mice underwent cranial window surgery completed according to previously published protocols without a port injection [54]. Following surgery, mice were returned to their home cage. Beginning at 3 months post-injection, this group of mice underwent long-term in vivo multiphoton imaging as described below. Following long-term imaging, all mice were euthanized by administration of isoflurane to induce a deeply anesthetized state, followed by decapitation and preparation for IHC as described below.

### Injection material

Untagged pre-formed fibrils of alpha-synuclein (PFF) injections were prepared according to previously published protocols [47, 53]. Briefly, fibrils were prepared in reactions (200 µl per tube) containing 360 µM (5 mg/ml) alpha-synuclein monomer in assembly buffer (50mMTris/100mMNaCl, pH 7.0). Reactions were incubated at 37 °C with constant agitation (1000 rpm) in an orbital mixer. Reactions were stopped after 5 days, aliquoted, and stored at 80 °C until use. PFF preparations were diluted (2 mg/ml) into sterile PBS and sonicated briefly before injection. mRuby-tagged PFF were made using recombinant mouse aSyn. mRuby was expressed from a PRK172 plasmid encoding wildtype mouse aSyn cDNA with a mRuby tag at the C-terminus, joined by a poly-His (6×) linker. The construct was expressed in BL21 (DE3) RIL E.coli cells and protein purified using a nickel affinity resin (Ni–NTA agarose; Qiagen) as per manufacturer’s instructions. The product was concentrated using Amicon Ultra filters (30 kDa cutoff; Millipore). Purity was verified by Coomassie staining following separation on a 12% SDS-PAGE gel. PFFs were assembled as previously described [44] and pathogenicity confirmed in wildtype mouse hippocampal neuron cultures.

### Pathology induction

*Port*-*injection animals* 6 (4 Female, 2 Male) A53T SynGFP mice were injected with the mouse WT sequence PFFs described above through pre-drilled holes in the previously placed cranial window between 2 and 5 months post-surgery (mean time of injection post cranial window surgery 2.5 months). Mouse WT sequence PFFs were chosen because, in our experience, they are robust producers of Lewy pathology in a range of WT and transgenic mouse lines. This is also true of our experience in A53T alpha-synuclein transgenic animals and may be related to the fact that the WT amino acid in mouse alpha-synuclein at position 53 is a threonine, not alanine, as it is in humans. Anesthetized animals (isoflurane 1% to 2%) were injected with 2.5 μl (2 mg/ml) of freshly sonicated PFFs using a stereotactic injection into right hemisphere primary sensory-motor cortex (1, −1.5, 0.3 mm). Following injection, animals were returned to their home cage. At 6 days post-injection all animals underwent in vivo multiphoton imaging over consecutive days, as described below. Following imaging, they were euthanized as described above and prepared for IHC as described below. This modified port-injection procedure was used to allow for immediate in vivo imaging after PFF injection in these animals. As a result of the port-injection procedure, the number and location of the specific areas visualized differed from mouse to mouse and was based on the initial injection site and the clarity of the individual cranial windows. Total number of areas imaged, total number of inclusions per animal, average number of inclusions per area and average density of inclusions per area can be found in Additional file 2: Table S2.

*Long*-*term*-*injection animals* A total of 12 (5 Female, 7 Male) A53T SynGFP mice were injected with mouse WT sequence PFFs according to our previously published protocols [54]. Anesthetized animals (isoflurane 1% to 2%) were injected with 2.5 μl (2 mg/ml) of freshly sonicated PFFs using a stereotactic injection into right hemisphere primary sensory-motor cortex (1, −1.5, 0.3 mm). Following injection, animals were returned to their home cage. Beginning at 2 months post-injection, a subgroup of 3 mice (1 Female, 2 Male) underwent cranial window surgery and long-term in vivo multiphoton imaging as described above. At variable and progressive time-points post-injection, all animals were euthanized by administration of isoflurane to induce a deeply anesthetized state, followed by decapitation and preparation for IHC as described below. An additional 4 A53T SynGFP mice (2 female, 2 male, age 5 m) did not receive injections and were used as control animals to determine baseline GFP expression in transgenic A53T SynGFP animals by cell-type.

*Peripheral IM injection animals* A total of 8 A53T SynGFP mice were injected with mouse WT sequence PFFs into the right gastrocnemius muscle between the ages of 2–3 months old. Anesthetized animals (isoflurane 1% to 2%) were injected with 5 μl (2 mg/ml) of freshly sonicated PFFs. Following injection, animals were returned to their home cage. At either 4- or 8-months post-injection animals were randomly selected for sacrificed as described above and prepared for IHC as described below.

*EM/CLEM animals* A total of 14 (9 female, 5 male) A53T SynGFP mice were injected with mouse WT sequence PFFs or alpha-synuclein monomer according to our previously published protocols [54]. Anesthetized animals (isoflurane 1% to 2%) were injected with 2.5 μl (2 mg/ml) of freshly sonicated PFFs using a stereotactic injection into right hemisphere primary sensory-motor cortex (1, −1.5, 0.3 mm; 3 female). An additional group of animals was injected with 2.5 μl (2 mg/ml) of freshly sonicated PFFs or 2.5 μl alpha-synuclein monomer using a stereotactic injection into the right striatum (−0.5, −2.0, 2.6 mm; 6 female, 5 male). Following injection, animals were returned to their home cage. Mice were then euthanized by overdose of anesthesia followed by perfusion and prepared for either transmission immuno-EM (striatal injection, 1 or 9-months post-injection) or CLEM (cortical injection 1-month post-injection) as described below.

### Mouse brain in vivo imaging and analysis

In vivo imaging and analysis was completed using a previously published protocol [54]. In the port-injection animals, A53T SynGFP aggregation was tracked in individual cells immediately following PFF injection. In long-term-injection animals, A53T SynGFP aggregation was tracked in individual cells beginning 3 months post-injection. All animals were imaged while anesthetized under isoflurane, using a Zeiss LSM 7MP multiphoton microscope outfitted with dual channel BiG (binary GaAsP) detectors and a Coherent Technologies Chameleon titanium-sapphire femtosecond pulsed laser source (tuned to 860 nm for imaging A53T SynGFP). Zeiss ZEN Blue image acquisition software was used. Images were analyzed with Fiji [66]. Briefly, regions of interest (ROIs) were selected for each cell in each collected imaging stack. Each individual cell was tracked overtime using the ROI and surrounding morphological features. Within each cell, the location of initial A53T SynGFP aggregation and the time course of inclusion formation was recorded. Data were analyzed in Prism 8 (GraphPad) to determine density of inclusions within each image stack (mm^3^) over time, % of total inclusions formed over time, and inclusion survival rate.

### Mouse brain removal, fixation, and sectioning

Mouse brains were dissected immediately postmortem, placed into vials of 6 ml of fresh 4% PFA in PBS, and fixed with a Pelco Biowave Pro for 90 min at 150 W at 30 °C in a circulating water bath. The brains were moved to 4 °C to continue to fix overnight. The next day the PFA was replaced with 0.05% sodium azide in PBS and tissue was stored at 4 °C until further processing. After fixation, the brains were sliced into 50 µm coronal floating sections using a Vibratome Leica VT1000S.

### Immunohistochemistry

Tissue slices were blocked for 1 h in blocking buffer (0.1% Triton-X, 10% goat serum, in PBS). Primary antibody was diluted in incubation buffer (1:5 dilution of blocking buffer) at a concentration optimized for each antibody and incubated overnight, in the dark, while shaking at room temperature. The tissue was washed for 30 min with PBS 5 times. The complementary secondary antibody was diluted in incubation buffer and incubated similarly overnight at room temperature. The next day, the tissue was washed with 5 exchanges of PBS. DAPI staining was done just prior to the final wash. The tissue was mounted onto a slide in CitiFluor CFMR2 Antifadent Solution. A #1.5 coverslip was sealed over the tissue with Biotium CoverGrip Coverslip Sealant. Antibodies and concentrations listed in Additional file 1: Table S1.

### Human neuropathological analysis

Human neuropathological analysis was done using standard histologic methods. Immunohistochemistry was used to evaluate serine-129-phosphorylated alpha-synuclein, Syn303, Syn505, and ubiquitin. In brief, formalin-fixed, paraffin-embedded sections of frontal cortex were incubated with antibody developed with diaminobenzidine (DAB) chromagen, and counterstained with hematoxylin, as previously described [40]. Antibodies and concentrations listed in Additional file 1: Table S1.

### Western blotting

Brains from male WT SynGFP [61], E46K SynGFP and A53T SynGFP that were 3–4 months of age were dissected immediately post mortem and olfactory bulbs and cerebellum removed. Remaining brain tissue was homogenized in ice-cold lysis buffer (50 mm Tris–HCl, pH 7.5, 5 M guanidinium, protease inhibitor mixture (Roche Complete)) using a probe-tip sonicator, then centrifuged (13,000 × *g*, 10 min) and supernatant was stored at −80 °C until analysis. On the day of analysis total protein concentration was determined using a BCA assay. Total cell lysates (12 μg of protein) were solubilized in LDS (lithium dodecyl sulfate) buffer under reducing conditions. Proteins were separated by SDS-PAGE using a 12% Bis–Tris gel and transferred to a PVDF membrane. Following blocking with blocking buffer (LI-COR Biosciences), membranes were incubated overnight with primary antibodies (alpha-synuclein: 1:1000, Syn-1, BD Biosciences; GAPDH: GAPDH, 1:10,000, Millipore). A near infrared fluorescent-labeled secondary antibody (1:5000; IR800CW; LI-COR Biosciences) was used and quantification was done with an Odyssey CLx infrared imaging system (LI-COR Biosciences) and ImageJ/Fiji (NIH).

### Electron microscopy

Electron microscopy (EM) was done similarly to previously published protocols [57, 86]. Animals were perfused using a transcardiac approach with 0.5% para-formaldehyde/1% glutaraldehyde/0.1% picric acid in 0.1 M phosphate buffer, pH 7.3, at room temperature. Tissue sections from both the primary sensory-motor cortex and the striatum were processed for EM pre-embed DAB immunolabeling for localization of p-129 alpha-synuclein using a microwave procedure. Tissue was incubated in the microwave (Pelco BioWave Pro) for 5 min, 550 W, at 35 °C with the vacuum off (all the remaining steps occurred at this temperature) in 10 mM sodium citrate, pH 6.0 (antigen retrieval), rinsed in 0.1 M phosphate buffer (PB) for 2 × 1 min at 150 W with the vacuum off, exposed to 3% hydrogen peroxide at 150 W for 1 min with the vacuum on, rinsed in PB at 150 W for 2 × 1 min with the vacuum off, exposed to 0.5% Triton X-100 for 5 min, 550 W with the vacuum on, washed in PB for 2 × 1 min at 200 W with the vacuum off, then exposed to the primary antibody for 12 min at 200 W 4 times using the following cycle: 2 min on, 2 min off, 2 min on, 5 min off, all on a continuous vacuum. The tissue was then rinsed in PB twice at 1 min each at 150 W with the vacuum off, and then exposed to the secondary antibody for 15 min at 200 W for 2 cycles of the following: 4 min on, 3 min off, 4 min on, 5 min off, all on a continuous vacuum. The tissue was then rinsed in PB, 2 × 1 min, at 150 W with the vacuum off and then exposed to ABC (Vector Elite Kit, 1 µl/ml solution A and B in PB) for 11 min at 150 W under vacuum using the following cycle: 4 min on, 3 min off, 4 min on. The tissue was then rinsed in PB twice at 1 min each, at 150 W with the vacuum off and then exposed to DAB (0.5 µg/ml + 1.5% hydrogen peroxide) for up to 10 min at room temperature. Thin Sects. (60 nm) were cut on an ultramicrotome (EMUC7; Leica) along the leading edge of the cortical tissue block, where layers I-VI were exposed, using a diamond knife (Diatome). Photographs (10/animal) were taken on a JEOL 1400 transmission electron microscope from a single 50 mesh grid (1 thin section/grid, 1 photograph/grid square) throughout the neuropil of layers II and III by an individual blinded to the experimental groups, using a digital camera (Advanced Microscopy Techniques). Antibodies and concentrations listed in Additional file 1: Table S1.

### Correlated light and electron microscopy (CLEM)

Tissue sections were prepared using OHSU’s Multiscale Microscopy Core (MMC) protocols and imaged using the MMC’s FEI CorrSight and Helios microscopes, and staff. A total of 3 PFF injected A53T SynGFP mice were perfused with 4% PFA in PBS and 0.2% glutaraldehyde at 1-month post-injection. Whole brains were post-fixed overnight in 4% PFA. Whole brains were then cut into 60 µm sections using a Vibratome. The injection site was carefully marked in each brain to localize the sections with the most abundant Lewy pathology. Unstained sections were then examined using a wide-field fluorescent microscope to locate and isolate sections with visible pathology. Areas of visible pathology were trimmed from larger sections into approximately 1 mm^2^ pieces. Individual squares of tissue were then placed in 5 mm glass vials for embedding. Sections of tissue were incubated in 0.5 M Tris–HCl pH 7.2, 0.1 M glycine at 4 °C for 2 h followed by 3 washes in PBS pH 7.2 on ice for 2 min each. Tissue sections then underwent a dehydration step down protocol (incubated for 5 min each at 30%, 50%, 75%, and 90% EtOH) on ice. Tissue sections were then incubated in a solution of 90% EtOH:London Resin White (LR White) at a 1:1 ratio for 1 h, followed by a 1:3 ratio for 1 h on ice. After this step, the tissue was infiltrated in 100% LR white overnight at −20 °C. On the following day the samples were exchanged with fresh cold LR White two times, 1 h each. Individual tissue sections were embedded in LR White in a wheaton cap and kept under vacuum at 50 °C for 24 h. Following embedding, individual sections were sectioned at 350 nm using a Histo Diamond knife (Diatome) and mounted on ITO coverslips. Prior to mounting ITO coverslips were ionized through a glow discharge negative set at 15 mA for 20 s using an EasiGlow unit (Ted Pella). Individual sections on ITO coverslips were imaged using the CorrSight™ using MAPS software to collect fluorescent images and determine the location of ROIs for EM imaging. Once all fluorescent images were collected, tissue was counterstained with 5% (w/v) uranyl acetate, Reynolds lead citrate and imaged using the Helios Nanolab 660. CLEM Images were collected and analyzed using the MMC’s software packages. Specifically, the presence and location of A53T SynGFP pathology was analyzed by surveying twenty 1 × 1 mm serial sections from each animal. Tissue sections were examined to determine the cellular and sub-cellular location of A53T SynGFP pathology. Control animals were used to determine background levels of fluorescence and response to injection. Electron micrographs were acquired in back scattered electron mode using a retractable directional backscattered (DBS) electron detector. Imaging conditions used were 1–1.5 keV, 100–200 pA and 4 mm working distance.

### Image acquisition

Fluorescent images were acquired on an Elyra-Zeiss Super-Resolution Microscope with AiryScan using a 63 × 1.4 NA oil objective. The lasers used were 561, 488, 405 and 647. Laser power was optimized for each laser and each round of immunohistochemistry and ranged from 0.3 to 2%. Master gain was optimized for each laser and each round of immunohistochemistry but ranged from 500 to 700. Images were analyzed using ImageJ/Fiji (NIH).

### Baseline GFP expression in transgenic A53T SynGFP animals by cell-type

**Fig. 1:**
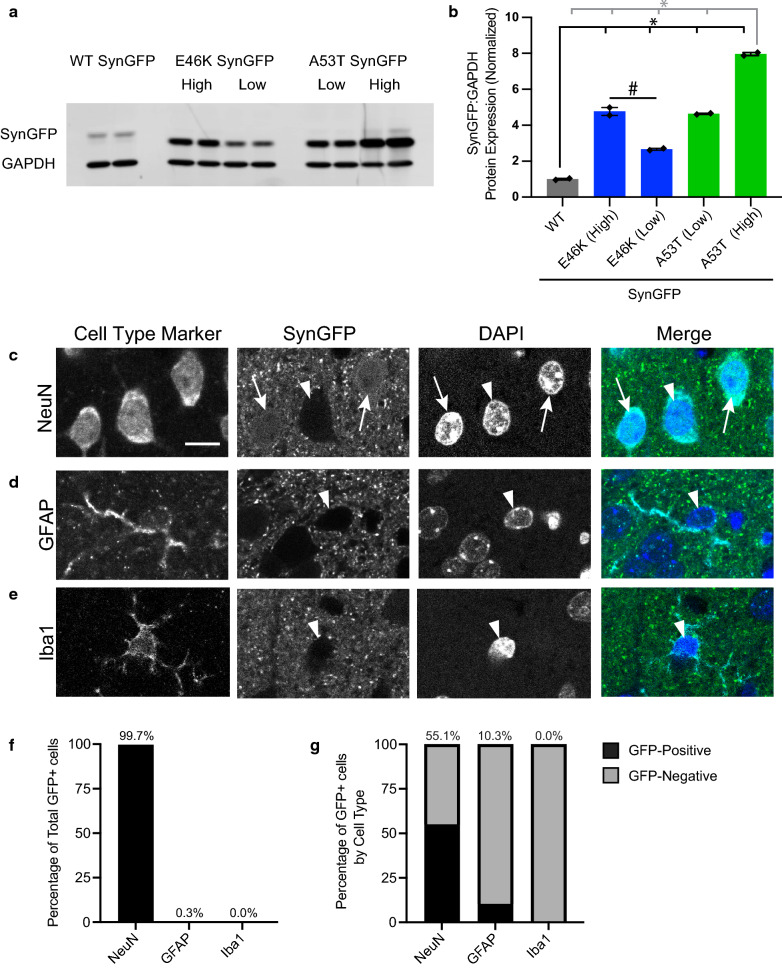
A53T SynGFP mice overexpress alpha-synuclein and the transgene localizes to neurons. **a** A53T SynGFP mice were generated by cloning the human SNCA gene, containing a point mutation at nt157 G > A causing an Ala53Thr change, into the MoPrp.Xho vector putting it under control of the murine prion promotor. SynGFP expression levels were determined by Western blot of brain lysate in WT and A53T (High) mice. Original data from Western blot also shows additional mouse lines that were not used in this study, including the A53T SynGFP (Low) line and the E46K SynGFP (High and Low) line. This blot was stained with antibodies to alpha-synuclein and GAPDH (loading control). Differences in apparent sizes of WT and mutant SynGFP proteins are due to the different linker regions used between alpha-synuclein and GFP. **b** Quantification of Western blot. SynGFP expression levels determined by Western blot of brain lysate indicate an overexpression of A53T SynGFP in this mouse line (High) of ~ 8-fold compared to a previously characterized human wild-type alpha-synuclein-GFP mouse line (WT SynGFP). Group data shows a significant difference in the normalized protein expression in the different transgenic mouse lines (one-way ANOVA (interaction F(4,5) = 570.1), p < 0.0001; Tukey’s multiple comparisons test, A53T (High) protein expression is greater than all other mouse lines: p < 0.0001 for all 4 comparisons, A53T (Low) protein expression is greater than E46K (Low) and WT: p = 0.0003 and p < 0.0001 respectively, E46K (High) protein expression is greater than E46K (Low) and WT: p = 0.0002 and p < 0.0001 respectively, and E46K (Low) protein expression is greater than WT: p = 0.007; A53T (Low) protein expression is not significantly different from E46K (High): p = 0.9047; N = 2 animals per line. **c**–**e** In A53T SynGFP transgenic animals, protein expression of the transgene was present in punctate staining in the neuropil and in relatively homogenous staining in a subset of cell bodies. Characterization of the cell types expressing SynGFP was determined using immunohistochemistry (IHC) and demonstrated that SynGFP is detectable in a subset of NeuN-positive cells (arrows), but rarely found in GFAP-positive or Iba1-positive cells. Arrow heads show SynGFP-negative cells. Scale bar equals 10 µm. **f** The majority of A53T SynGFP positive cells were NeuN-positive (99.7%), while a very small fraction of A53T SynGFP positive cells were GFAP-positive (0.3%), and none (0.0%) were Iba1 positive (N = 815 cells). **g** Of the total NeuN-positive cells, 55.1% were GFP positive, indicating expression of the A53T SynGFP transgene in a large fraction of this cell type population. 10.3% of GFAP-positive cells were GFP positive, and no Iba1-positive cells were GFP positive. Based on this characterization, expression of the A53T SynGFP transgene is significantly increased in neuronal cells, rather than non-neuronal cells, in these animals (Chi-square (2) = 106.4, p < 0.0001, N = 1553 cells)

GFP expression in the cell was identified by two blinded reviewers and scored as positive or negative for GFP expression. See Fig. 1c–e to reference cells that are GFP-positive or negative. The cell-identifying channel was removed during scoring to ensure that both independent reviewers were blinded to cell identity when scoring GFP signal. Every GFAP and Iba1 cell in the data set was counted, n = 29 and 75 respectively, and 8% of the NeuN cells were counted, n = 109 of 1449 total. To estimate the total number of NeuN cells that were GFP positive in the entire data set, the percentage of GFP-positive NeuN cells was multiplied by the total number of NeuN cells.

### Pathology analysis following peripheral IM PFF injection

The overall number and location of cells and neurites with A53T SynGFP pathology was quantified in serial sections of mouse brain spanning from the caudal brainstem (Bregma −2.69 mm), to the midbrain (Bregma −2.27), to the motor cortex (Bregma −1.07) at both 4 month and 8 month post-injection time points (Fig. 7g). The amount of pathology in specific brain areas was counted and compared in each section at each time point to determine the location and time course of spread. Data was analyzed in Image J/Fiji (NIH) using plugins to threshold, process noise, and analyze particles with control (c) and motor (m) regions of interest.

### Experimental design and statistical analysis

All quantified values are reported as the mean ± SEM, unless otherwise noted. The relevant sample type and number (N), and statistical tests used to evaluate significance for each experiment are presented in the figure legend of each data set. The sample sizes used in each experiment were based on estimates of expected effect sizes (~ 10–50%) and standard deviations from preliminary data and were powered to detect differences with a 0.05 significance (α) level and 0.9 power (1-β). Potential sex differences were also analyzed in our experiments and none were detected.

## Results

### A53T SynGFP mice overexpress alpha-synuclein and the transgenic protein localizes primarily to neurons

Experiments in this study were carried out using an alpha-synuclein transgenic mouse model we recently developed. This model overexpresses the human A53T point mutation alpha-synuclein tagged on its C-terminus with enhanced green fluorescent protein (A53T SynGFP) under control of the mouse prion promotor. The expression level of A53T SynGFP in this new mouse line was determined using western blot and compared to a previously well-characterized human wild-type alpha-synuclein-GFP mouse line, which shows a ~ 3-fold over-expression compared to human brain (WT SynGFP) [19, 54, 61, 76, 83]. Our results showed an ~ 8-fold increase in expression of SynGFP in the new A53T SynGFP mice compared to the WT SynGFP line (~ 24-fold over-expression compared to human brain, Fig. 1a, b). To further characterize this new mouse line, IHC was used to determine the location of transgenic protein expression. Staining showed that A53T SynGFP signal is present in neuropil puncta and as a homogenous signal in a subset of cell bodies (Fig. 1c–e). Using CLEM and immuno-EM techniques, we found that neuropil puncta staining reflects A53T SynGFP localization to presynaptic terminals (Additional file 4: Fig. S1). Known cell type markers were used to determine the specific cell type that co-localized with homogenous A53T SynGFP somatic signal in the cortex and showed that A53T SynGFP localizes mainly to cells positive for the neuronal marker NeuN (99.7% of GFP positive cortical cells are NeuN positive), with little to no protein expression found within cells positive for an astrocyte marker (GFAP, 0.3% of cortical cells) or a microglia marker (Iba1, 0.0% of cortical cells) (Fig. 1f). Additional analyses showed that over half of all the NeuN positive cells were A53T SynGFP positive (55%), while only 10% of the GFAP positive cells were A53T SynGFP positive, and no Iba1 positive cells were A53T SynGFP positive (Fig. 1g). Thus, we have found that A53T SynGFP localizes primarily within neurons in the cortex in this mouse line.

### Electron micrographs show fibrillar structures that co-localize with SynGFP fluorescent signal using CLEM imaging

**Fig. 2:**
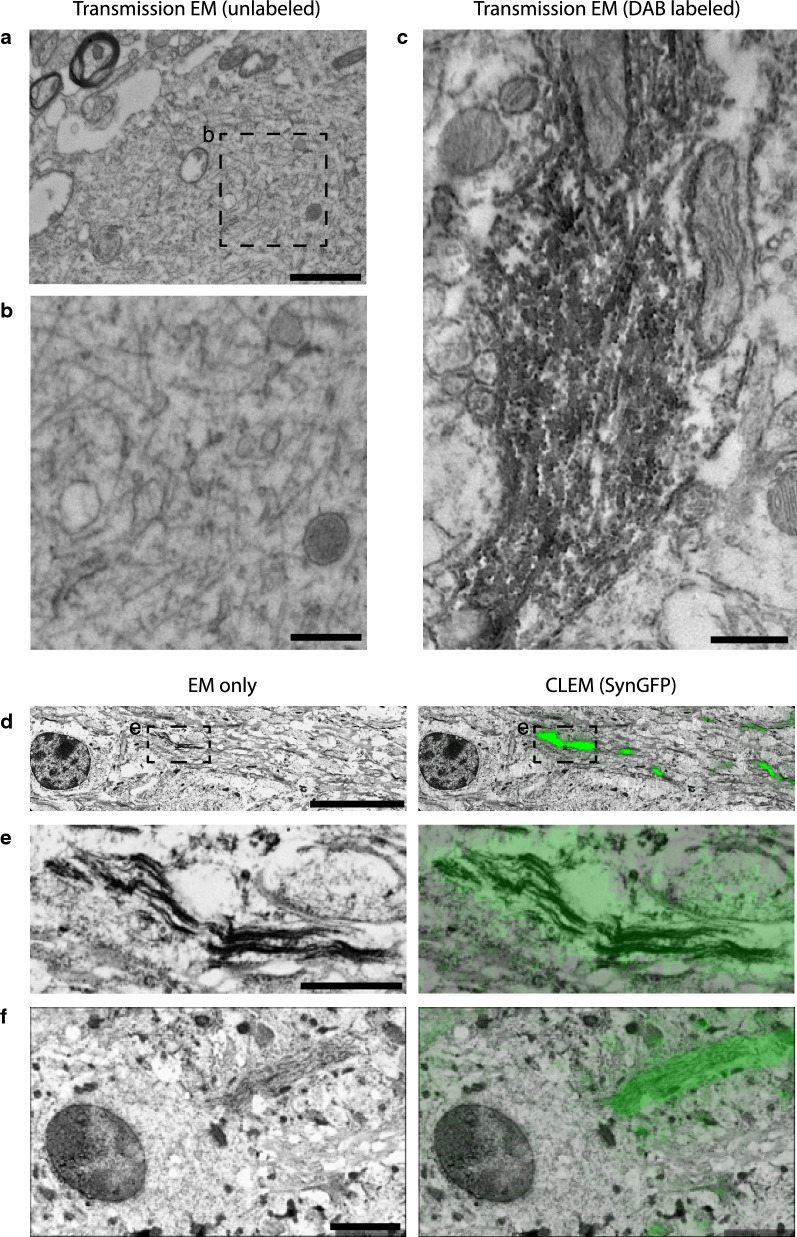
Electron Micrographs show fibrillar structures that co-localize with SynGFP fluorescent signal using CLEM imaging. **a** Transmission Electron Microscopy (TEM) image of a large neurite from the motor cortex of a SynGFP mouse after PFF injection. Scale bar 1500 nm. **b** Inset demonstrating an example of fibrillar structures within the neurite. Scale bar 500 nm. **c** DAB/p-129 alpha-synuclein labeled neurite in the striatum of a SynGFP mouse after PFF injection. DAB labeling is consistent with disordered alpha synuclein fibrillar structures as opposed to the more organized appearance expected in microtubules. Scale bar 500 nm. **d** Left panel: Electron Microscopy (EM) image of a cell and white matter tracks from the motor cortex of a SynGFP mouse 1-month after PFF injection. Right panel: The same EM image with an overlay of the fluorescent SynGFP signal captured from the same location using MAPS software creating a Correlated Light and Electron Microscopy (CLEM) image. Scale bar 5 µm. **e** Left panel: EM image of the inset from Fig. 2d demonstrating an example fibrillar structure within white matter tacks. Right panel: CLEM image showing co-localization of the fluorescent SynGFP signal with the fibrillar structure. Scale bare 1 µm. **f** Left panel: EM image of a neuron in the motor cortex of the same SynGFP mouse 1-month after PFF injection depicting a fibrillar structure within a neuritic process. Right panel: CLEM image showing co-localization of the fluorescent SynGFP signal with the fibrillar structure. Scale bar 2 µm

We performed transmission electron microscopy and CLEM imaging on A53T SynGFP mice following PFF injection to test if Lewy pathology could be seeded in these mice and to determine the ultrastructural characteristics of these inclusions. Tissue was examined using transmission electron microscopy techniques 9 months after PFF injection into the striatum. Fibrillar structures were found to be present in neuritic structures in the motor cortex (Fig. 2a, b) and were also labeled with DAB/p-129 alpha-synuclein immunostaining (Fig. 2c) within the striatum. However, it was not easily possible to determine if these p-129-positive fibrillar structures were composed of A53T SynGFP or endogenous mouse alpha-synuclein using transmission EM techniques. Tissue was then examined in a separate group of animals, 1 month after PFF injection into the primary sensory-motor cortex, where fixation was optimized for CLEM imaging. A region of interest with fluorescent A53T SynGFP inclusions near the injection site was dissected out and prepared for CLEM analysis. EM results show that fibrillar material is present in neuritic structures and perfectly overlays with SynGFP fluorescence (Fig. 2d–f), establishing that these inclusions are composed of A53T SynGFP in a fibril state.

### A53T SynGFP mice form Lewy Body inclusions at an accelerated rate following PFF injection and results in rapid neuronal cell death

**Fig. 3:**
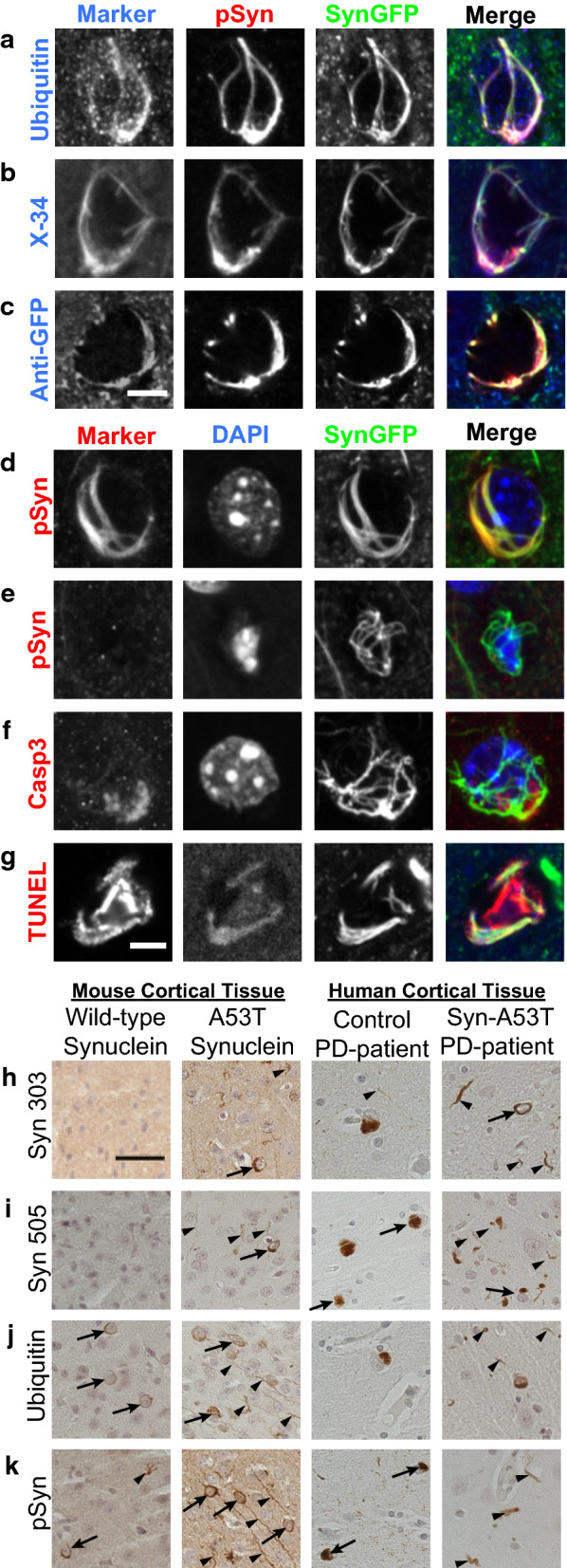
A53T SynGFP inclusions co-localize with classical hallmarks of human Lewy pathology. **a**–**g** Mature A53T SynGFP inclusions are phosphorylated at serine-129 (pSyn), ubiquitinated, in an amyloid dye-binding configuration (X-34), co-stain with an anti-GFP antibody, and a small subset stain positively for cell death markers (Casp3, TUNEL). There was also a small subset of inclusions that were A53T SynGFP positive but did not stain for pSyn (e), and often these inclusions show signs of undergoing cell death processes as evidenced by their small, condensed nuclei (DAPI in e). Scale bar 5 µm. **h**–**k** A53T SynGFP inclusions also share the characteristics of Lewy pathology found in human genetic cases with the A53T mutation. Tissue from PFF-injected A53T mouse samples and human clinical samples contain somatic (arrow) and neuritic (arrow head) inclusions that stain with alpha-synuclein antibodies that selectively detect pathologic synuclein (Syn303 and Syn505), are ubiquitinated, pSyn positive, and increased in number compared to both mouse wild-type and human control PD-patient tissue. Scale bar 50 µm

To further characterize the nature of alpha-synuclein inclusions and compare them to established markers of human Lewy pathology, A53T SynGFP mice were injected with mouse WT sequence PFFs using stereotactic injection into right hemisphere primary sensory-motor cortex. At various time points post-injection, IHC analysis showed bilateral cortical pathology in PFF injected A53T SynGFP mice that was morphologically consistent with Lewy pathology and co-localized with classical markers, including phosphorylation at serine-129 (pSyn), ubiquitination, and staining with amyloid-binding dyes (Fig. 3a–g). In addition, PFF-induced inclusions co-stained with an anti-GFP antibody, indicating that the green fluorescence we observe comes from the transgenic protein and not endogenous auto-fluorescent species (Fig. 3c). As a control, we also performed a set of PFF injections into transgenic mice that only express GFP (Thy1-GFP, M-line). In these mice, neurons formed Lewy pathology composed of endogenous mouse alpha-synuclein, however, GFP expression in such neurons did not colocalize well with Lewy pathology (Additional file 5: Fig. S2). This indicates that GFP by itself is not recruited to Lewy pathology unless it is fused to alpha-synuclein, as it is in WT SynGFP [54] and A53T SynGFP mice. A small subset of inclusions stained positively for cell death markers indicating active neurodegeneration (Fig. 3f, g, Additional file 6: Fig. S3). An additional small subset of A53T SynGFP-positive inclusions did not stain for pSyn (Fig. 3e), and these inclusions showed signs of undergoing active cell death, including containing small, condensed pyknotic nuclei by DAPI staining (Fig. 3e). Although A53T SynGFP inclusions were rarely found in spherical inclusions characteristic of Lewy bodies, when compared to Lewy pathology found in human genetic cases with the A53T mutation, a more striking resemblance was noted (Fig. 3h–k). Tissue from PFF-injected A53T SynGFP mouse samples and A53T human samples both had flame-shaped somatic and neuritic inclusions that were immunoreactive for pathologic alpha-synuclein (Syn303; Syn505). Inclusions were also ubiquitinated, pSyn-positive, and numerous in cortical regions, further demonstrating that A53T SynGFP inclusions are pathologically relevant (Fig. 3h–k). We similarly did unilateral striatal PFF injections in another set of A53T SynGFP animals and analyzed their brains at 1 month and 9 months post-injection. The 1-month post-injection animals showed no obvious spread of Lewy pathology outside of a limited amount near the injection site in the striatum (data not shown). In contrast, 9 months post-injection we saw significant spread of Lewy pathology in the ipsilateral (Additional file 7: Fig. S4) and contralateral hemispheres (data not shown).

**Fig. 4:**
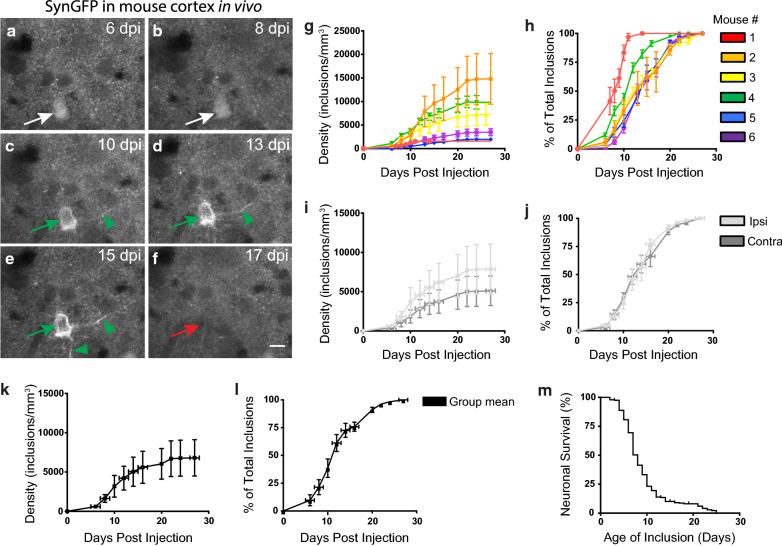
A53T SynGFP mice form Lewy Body inclusions at an expedited rate following PFF injection and show a rapid cell death time course. **a**–**f** In vivo imaging of individual cells over time shows rapid inclusion formation and degeneration of inclusion-bearing cells. Example images show an individual cell tracked over time (arrows). The cell was followed beginning at 6 dpi and shows SynGFP fluorescence in puncta at terminals and homogeneous fluorescence in the cell body indicating soluble SynGFP (white arrows). At 10 dpi the inclusion begins to form and SynGFP fluorescence starts to clear from the nucleus and become more intense at the edges of the cytoplasm, indicating aggregated SynGFP (green arrows). At 15 dpi SynGFP aggregation is clearly visible in the processes of the cell (arrow heads). Within a 2-day window the inclusion disappears and at 17 dpi the inclusion-bearing cell has degenerated (red arrow). Survival time of this inclusion-bearing cell was 7 days. Scale bar 10 µm. **g**–**l** A53T SynGFP inclusions form at a much faster rate compared to previously published data tracking inclusion formation in WT SynGFP mice. A total of six A53T SynGFP mice were tracked in this study (1–6). Individual cells were tracked overtime in cortex layer II/III of both the injected (ipsi) and un-injected (contra) hemispheres. In all mice mature SynGFP inclusions started forming within 10 days post injection (dpi) compared to the 3–4 months post injection (mpi) time frame previously reported in WT SynGFP mice. Formation rate was tracked as both a measure of overall density (inclusions/mm^3^) (**g, i, k**) and as a percentage of total inclusions formed (**h, j, l**). Individual mice (**g, h**), group data as a function of location (ipsi, contra) (**i, j**) and overall group data (**k, l**) and are shown. A53T SynGFP mice show both intra and inter-animal variability in the density and percentage of inclusions formed at each time point (SEM), but the overall rate of formation followed the same rapid time course in all mice. **m** Group data shows rapid degeneration of inclusion-bearing cells, with a median survival time of 8 days after inclusion formation

Using similar in vivo imaging and analysis techniques as we previously used for WT SynGFP mice [54], we were able to follow individual cells over time to determine the rate of inclusion formation and cell death (following inclusion formation) after PFF injection in A53T SynGFP mice (Fig. 4a–f). We found that the A53T SynGFP mice developed bilateral cortical pathology rapidly (Fig. 4g–l, Additional file 1: Table S1). A53T SynGFP inclusions started forming within 10 days post-injection, compared to the 3–4 month post-injection time frame we previously reported in WT SynGFP mice [54] or the 1 month time frame that others have shown in untagged alpha-synuclein WT or mutant transgenic models [29, 45, 46, 59, 60]. The inclusions that we detect with in vivo imaging are very likely composed of aggregated alpha-synuclein, since our previous work using in vivo multiphoton fluorescence recovery after photobleaching (FRAP) techniques clearly shows drastically decreased mobility of SynGFP within Lewy inclusions imaged in this way [54]. We also observed very rapid cell death after inclusion formation (Fig. 4m), since in this mouse model inclusion-bearing cells had a half survival time of ~ 8 days, as compared to a ~ 180 days half survival time in the WT SynGFP mouse line [54].

### A53T SynGFP aggregates appear first in axons

**Fig. 5:**
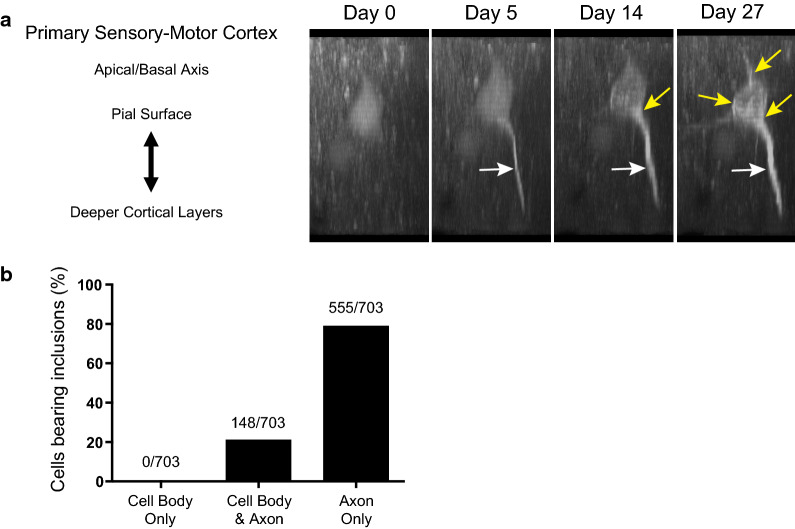
A53T SynGFP aggregates appear first in axons. **a** Live in vivo multiphoton 3D projection showing serial images of the same neuron at progressive time points after PFF injection. A53T SynGFP aggregates (white arrow) form first in the axon and predict formation of a somatic inclusion (yellow arrows) in this neuron. **b** Group data show the location of initial A53T SynGFP aggregate formation as a percentage of analyzed cells. The overwhelming majority of the time (555/703; 78.95%) the first evidence of SynGFP aggregation was in the axon alone. In a subset of cells (148/703; 21.05%) the first evidence of aggregation was in both the axon and the cell body, indicating that the initial aggregate formation was not captured at the specific imaging time point at which those cells were imaged. There was no evidence (0/703; 0.00%) that the initial site of aggregation was in the cell body. This is strongly suggests aggregation begins in axons (Chi-square (2) = 505.8, p < 0.0001, N = 703 cells)

The rapid time course of inclusion formation in A53T mice allowed us to capture the initial location of A53T SynGFP aggregation within individual cells. We used in vivo multiphoton imaging to follow individual cells prior to inclusion formation and acquired imaging volumes within cortex to analyze and track the movement of aggregated A53T SynGFP in specific structures starting before somatic inclusions formed. Careful analysis of the in vivo multiphoton imaging data shows that alpha-synuclein aggregates form almost exclusively in axonal structures and then propagate in a retrograde fashion to the soma (Fig. 5). The designation of axon was made based on the morphological features, direction, length, and depth of the neuritic process. Each cell was analyzed individually within a confocal volume that began at the pial surface of the brain and extended to a depth of about 200 µm into the cortex. In the majority of cells, the location of initial A53T SynGFP aggregation could be seen at early time points as a linear process oriented with its long axis perpendicular to the surface of the brain and running from deeper to more superficial layers (Fig. 5a, video from Additional file 8: Fig. S5). Branching of these initially labeled neuritic aggregates was never observed, suggesting that these neurites are unlikely to be dendrites. A complete analysis of over 700 cells revealed that ~ 80% of the time, at the first imaging session, an aggregated A53T SynGFP inclusion could be detected in the axon before the presence of a somatic inclusion in that cell. In ~ 20% of the cells at the first imaging session, inclusions were visualized in the axon and the cell body simultaneously, and there were no instances where inclusions were initially visualized in the cell body and then later progressed into the axon (Fig. 5b). We believe that in ~ 20% of cells, where the first imaging session demonstrated axon and cell body inclusions simultaneously, we are seeing a time point that represents cells where retrograde spread from axon to the soma had already occurred before we were able to capture the event using in vivo imaging. The fact that spread starting in the soma and spreading to the axon was never detected in > 700 cells studied suggests that if anterograde spread does occur, it must happen at a very low rate. Therefore, our in vivo data strongly suggest the axon is the initial location of inclusion formation after PFF injection into the primary sensory-motor cortex in A53T SynGFP mice.

In a subsequent analysis of fixed tissue, we co-stained with an axonal protein marker (SMI-312) and the somatodendritic marker (MAP2) to further explore the specific cell structure that contained neuritic inclusions (Additional file 9: Fig. S6). There was co-localization between the axonal protein marker and neuritic inclusions (Additional file 9: Fig. S6a). However, the complete analysis showed that only a small subset of the neuritic inclusions co-localized with the specific axonal protein marker that we tested (SM1-312). There was no evidence of co-localization with the somatodendritic marker MAP2. In this experiment, the majority of neuritic inclusions did not co-localize with SMI-312 or the somatodendritic marker (Additional file 9: Fig. S6b). Presumably this is because fibrillar SynGFP does not often co-localize with this specific axonal protein. Fluorescently-labeled mRubyPFFs (in contrast to the unlabeled PFFs used for the rest of our study) were also used to determine the initial location of uptake and aggregation in A53T SynGFP mice. At very early time points post-injection (1–4 weeks post-injection), we found that labeled PFFs are present along axons in deep subcortical white matter and progressively move away from the site of injection towards the corpus callosum (Additional file 10: Fig. S7). These data together strongly suggest that both labeled PFFs and aggregated A53T SynGFP move in a retrograde fashion from axons to cell bodies.

### A53T SynGFP inclusions form in non-neuronal cells at longer intervals post-injection

**Fig. 6:**
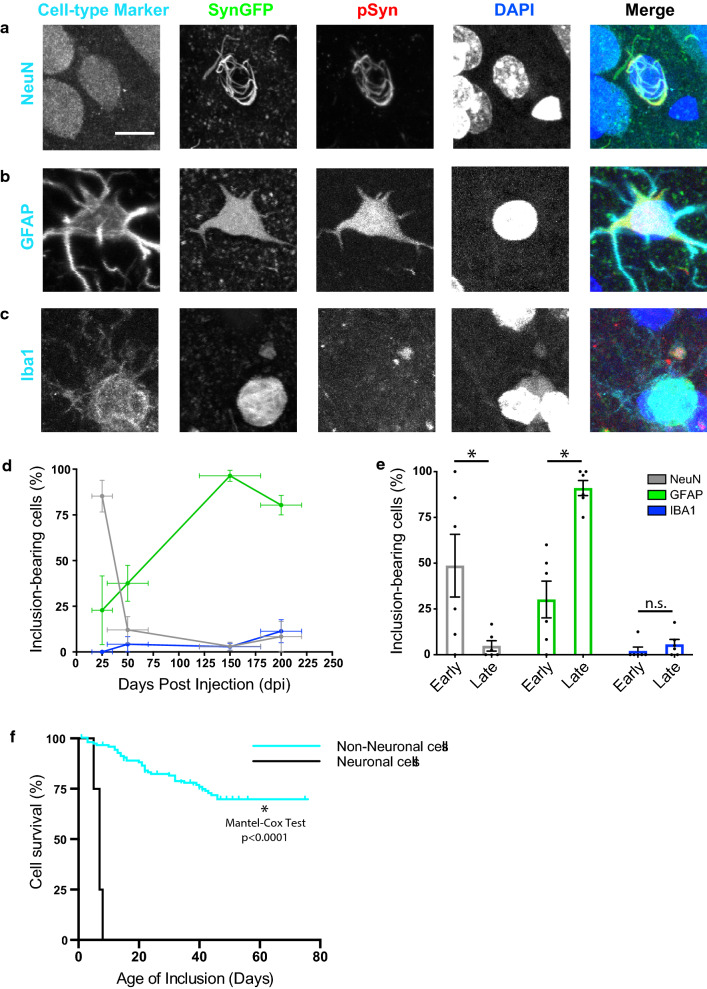
A53T SynGFP inclusions form in non-neuronal cells at longer intervals post-injection. **a**–**c** A53T SynGFP inclusions appear in at least three cell types: NeuN-positive cells (**a**), GFAP-positive cells (**b**), and Iba1-positive cells (**c**). The morphology and staining characteristics of these inclusions differed depending on cell-type identified via immunohistochemistry. For example, Iba1-positive SynGFP inclusions rarely co-stain with psyn (**c**). Scale bar 10 µm. **d** The percentage of A53T SynGFP inclusions in different cell types varies over time. At earlier intervals post-injection (less than 50 days post-injection (dpi)) A53T inclusions are more likely found in NeuN-positive cells than GFAP-positive cells or Iba1-positive cells, as identified via immunohistochemical analysis. At later intervals post-injection (greater than 50 dpi) A53T SynGFP inclusions are more likely found in GFAP-positive cells than Iba1-positive or NeuN-positive cells. Cells identified as Iba1-positive are unlikely to contain A53T SynGFP inclusions regardless of the post-injection interval, although a preference for the later interval is observed. NeuN = gray line, GFAP = green line, and Iba1 = blue line. **e** Group data shows that the cell-type dependence of A53T SynGFP inclusions varies in accordance with days post injection (dpi) (two-way ANOVA (interaction F(2,30) = 19.15), (Early vs. Late F(1, 30) = 0.9875), (cell type F(2,30) = 22.71), p < 0.0001, p = 0.3283, and p < 0.0001 respectively; Sidak’s multiple comparisons test, at early intervals there are more NeuN-positive inclusions bearing cells than at late intervals: p = 0.0029, at early intervals there are less GFAP-positive inclusion bearing cells than at late intervals: p < 0.0001, and there is no difference in the percentage Iba1-positive inclusions cells at the early and late timepoint: p = 0.9867; N = 6 animals per timepoint). Early intervals post-injection equal less than 50 days post-injection (dpi). Late intervals post-injection equal greater than 50 dpi. Mean and SEM of percentage of inclusions from each slice analyzed from each animal are shown at each time point. NeuN = gray, GFAP = green, and Iba1 = blue. **f** A53T SynGFP inclusion survival time is dependent on cell-type. In vivo imaging of individual cells over time showed variable rates of degeneration in inclusion-bearing cells. Based on morphology, neuronal and non-neuronal cell types were identified from in vivo data. Group data continues to show rapid degeneration of inclusions-bearing cells that are neuronal cells (median survival 7 days), however extended survival time is found in non-neuronal cells (data never reaches 50% survival); Mantel-cox test p < 0.0001. Neuronal cells = black, Non-neuronal cells = cyan

IHC analysis was performed on brain sections of mice at various times after PFF injection into the right hemisphere primary sensory-motor cortex. Results show that both neuronal and non-neuronal inclusions form following PFF injection in A53T SynGFP mice (Fig. 6a–c), but that the shift from neuronal to non-neuronal inclusions occurred in a time-dependent manner (Fig. 6d). At early time points (prior to 50 days post-injection) the majority of inclusions formed in NeuN-positive cells (17/33), while at later time points (after 50 days post-injection) the majority of inclusions formed in GFAP-positive cells (147/160). At both time points inclusions were unlikely to form in Iba1-positive cells (early = 1/24; late = 7/122), and when they did they had somewhat different properties (pSyn-negative SynGFP inclusions, Fig. 6e) potentially representing close association with or engulfment by microglia of degenerating Lewy-bearing structures. A secondary analysis was completed using the TMEM119 antibody, which is more specific to mouse microglia, and results were not significantly different from that produced with Iba1 staining (Additional file 11: Fig. S8).

### A53T SynGFP inclusion survival time is dependent on cell-type

A separate group of A53T SynGFP mice underwent PFF injection and long-term in vivo imaging was conducted to further characterize the pathological progression of Lewy pathology. Interestingly, the survival time of cells with A53T SynGFP inclusions was dependent on the cell-type in which the inclusion formed. Neuronal cells were separated from the non-neuronal cells in the in vivo imaging dataset based on morphological features. We found that neurons underwent rapid cell death similar to that observed previously, with half survival time 7–8 days (Figs. 4m, 6f). However, longer imaging showed that later appearing inclusions in non-neuronal cells survived much longer, with half survival time > 80 days (Fig. 6f).

### Peripheral intramuscular (IM) injection data shows a time-dependent and progressive spread of alpha-synuclein inclusions through neuroanatomically connected pathways

**Fig. 7:**
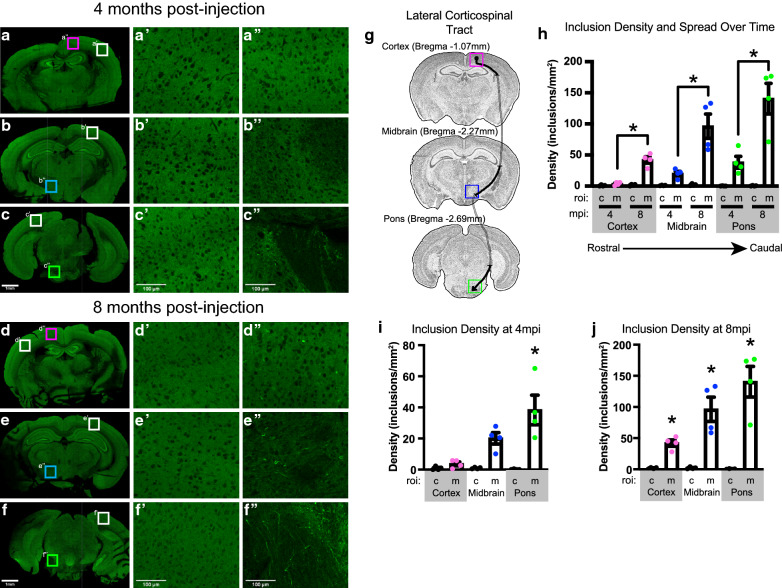
Peripheral intramuscular (IM) injection data showing time-dependent and progressive spread of alpha-synuclein inclusions through known neuroanatomically connected pathways. **a**–**c** A53T SynGFP inclusions appear specifically in motor regions of interest (ROI) in a time-dependent manner. Fixed tissue images of representative brain sections along the rostral-caudal axis. **a** Representative cortex section that includes the primary motor cortex (motor ROI, pink square) and the somatosensory barrel cortex (control ROI, white square). **b** Representative midbrain section that includes cerebral peduncle and the zona incerta (motor ROI, blue square) and the primary somatosensory area (control ROI, white square). **c** Representative pons section that includes cerebral peduncle and the medial lemniscus (motor ROI, green square) and primary visual cortex (control ROI, white square). At the 4-month time point, A53T SynGFP inclusions are visible in motor ROI in the pons (**c**″) and to a lesser extent in the midbrain (**b**″), but are not detectablef in the cortex (**a**″) or in any of the corresponding control ROI (**a**′–**c**′). Scale bar 1 mm in a–c and 100 µm in a′–c′ and a″–c″. **d**–**f** A53T SynGFP inclusions appear specifically in motor regions of interest (ROI) in a time-dependent manner. Fixed tissue images of representative brain sections along the rostral-caudal axis. **d** Representative cortex section that includes the primary motor cortex (motor ROI, pink square) and the somatosensory barrel cortex (control ROI, white square). **e** Representative midbrain section that includes cerebral peduncle and the zona incerta (motor ROI, blue square) and the primary somatosensory area (control ROI, white square). **f** Representative pons section that includes cerebral peduncle and the medial lemniscus (motor ROI, green square) and primary visual cortex (control ROI, white square). At the 8-month time point, A53T SynGFP inclusions are visible in all motor ROI along the rostral caudal axis (**d**″–**f**″) but are not visible in any of the corresponding control ROI (**d**′–**f**′). Scale bar 1 mm in **a**–**c** and 100 µm in **a**′–**c**′ and **a**″–**c**″. **g** Schematic showing the rostral to caudal locations of serial brain sections analyzed for each animal. Specific sections were chosen based on the presence of a motor region of interest (ROI). Motor ROI were determined based on known descending motor pathways in the cortex (pink square), midbrain (blue square), and pons (green square). The specific ROI were chosen because they encompass white matter tracts and nuclei within the lateral descending motor pathways, specifically the lateral corticospinal tract and the rubrospinal tract. These tracts begin in the primary motor cortex or red nucleus, respectively, and terminate in the lower motor neurons of the anterior gray horn of the spinal cord. Both tracts are connected to the site of PFF injection in the gastrocnemius muscle through multiple synaptic connections, thereby representing sites of retrograde trans-synaptic spread. **h** Quantification of SynGFP inclusions in each region of interest (ROI) at each time point (months post-injection (mpi). Density of inclusions in each ROI (mm^2^) was calculated in ImageJ using particle analysis. ROI equaled the motor (m) or control (c) area in each brain region (cortex, midbrain, pons) at each timepoint (4 vs. 8 mpi). Group data shows that the density of inclusions was significantly different in the motor ROI in all brain areas at 4 and 8mpi, but control ROI did not differ at 4 and 8mpi (one-way ANOVA F(11,138) = 43.55), p < 0.0001; Sidak’s multiple comparisons test, 4 vs. 8 mpi motor ROI (m): cortex, midbrain, pons p < 0.0001 for all 3, 4 vs. 8 mpi control ROI: cortex, midbrain, pons p > 0.9999 for all 3; N = 6–20 ROIs/3–4 animals per group. Mean and SEM of the density of inclusions in each group are shown in Additional file 3: Table S3. Cortex = pink, midbrain = blue, and pons = green. **i** At 4mpi there was a significant difference between the motor and control ROI in the pons alone, indicating trans-synaptic spread only to the most caudal brain section. Sidak’s multiple comparisons test, motor vs. control in cortex, midbrain, and pons: p > 0.9999, p = 0.1287, p = 0.0126, respectively. Mean and SEM of the density of inclusions in each group are shown in Additional file 3: Table S3. Cortex = pink, midbrain = blue, and pons = green. **j** At 8mpi there was a significant difference between motor and control ROI in each section along the rostral-caudal axis indicating progressive trans-synaptic spread through connected brain areas along the descending motor pathway following IM PFF injection. Sidak’s multiple comparisons test, motor vs. control in cortex, midbrain, and pons: p < 0.0001 for all 3. Mean and SEM of the density of inclusions in each group are shown in Additional file 3: Table S3. Cortex = pink, midbrain = blue, and pons = green

To test the hypothesis that alpha-synuclein inclusions spread through neuroanatomically connected pathways as opposed to other mechanisms, A53T SynGFP mice were injected with mouse WT sequence PFFs into the right gastrocnemius muscle between the ages of 2–3 months old. Animals were euthanized either at 4 months or 8 months post-injection. Brains were dissected, fixed, and serial sectioned from the forebrain, through the brainstem, to the most rostral section of the spinal cord. Sections from designated regions of interest through the rostral – caudal axis of the brainstem were chosen, and both motor and control regions of interest were analyzed (Fig. 7). Motor regions of interest were determined based on known descending motor pathways. The specific areas were chosen because they encompass white matter tracts and nuclei within the lateral descending motor pathways, specifically the lateral corticospinal tract and the rubrospinal tract (Fig. 7g). These tracts begin in the primary motor cortex or red nucleus, respectively, and terminate in the lower motor neurons of the anterior gray horn of the spinal cord. Both tracts are connected to the site of PFF injection in the gastrocnemius muscle through multiple synaptic connections, thereby representing potential sites of retrograde trans-synaptic spread. Results showed that A53T SynGFP inclusions are detectable specifically in motor areas in a time-dependent manner following PFF injection into the gastrocnemius muscle in the periphery. At 4 months post-injection, A53T SynGFP inclusions were detected in motor regions of interest in the pons and midbrain, but were not detectable in the cortex or in any of the corresponding control regions of interest (Fig. 7a–c). At 8 months post-injection, A53T SynGFP inclusions were detected in all motor regions of interest along the rostral-caudal axis studied, including motor cortex, but were not detected in any of the corresponding control regions of interest (Fig. 7d–f). Quantification of the number of inclusions in each region of interest showed a significant effect of region of interest on number of inclusions. Post-hoc multiple comparison testing showed a significant difference between all 3 motor regions of interest at 4 months compared to 8 months, with a greater number of inclusions present in each region of interest along the rostral caudal axis at 8 months compared to 4 months, indicating an increase in inclusion number over time (Fig. 7h). At 4 months post-injection a significant difference was seen between the motor and control areas in the pons alone, indicating trans-synaptic spread only to the caudal brainstem at the earlier time point (Fig. 7i). At 8 months post-injection, there was a significant difference between motor and control regions of interest in each location along the rostral-caudal axis, indicating progressive trans-synaptic spread through connected brain areas along the descending motor pathway following IM PFF injection (Fig. 7j). Thus, both a progressive and region-specific spread of A53T SynGFP inclusions was observed (Fig. 7h–j), providing support for the hypothesis that alpha-synuclein inclusions spread trans-synaptically through neuroanatomically connected pathways in a time-dependent manner.

## Discussion

We have developed a new transgenic mouse model of synucleinopathy that expresses human A53T SynGFP under control of the mouse prion protein promoter in a majority of cortical neurons. Our characterization and study of this line demonstrates that it exhibits several distinct advantages over other, currently available, mouse models. This model demonstrates very rapid development of PFF-induced alpha-synuclein pathology in the cortex and the subsequent accelerated cell death of inclusion-bearing cells (Fig. 4). This new model enabled rigorous investigation of the initial location of Lewy pathology formation and propagation in the living brain. These findings strongly suggest that aggregation begins in axonal structures with retrograde propagation to the cell body (Fig. 5). We also found that somatic aggregated alpha-synuclein inclusions developed rapidly in neurons. However, as time since PFF injection progressed, we saw a transition to inclusions forming in non-neuronal cells, which were predominantly astrocytes. These astrocytes survived much longer after inclusion formation than neurons (Fig. 6). This model also allowed us to study peripheral to central nervous system spread of Lewy pathology after PFF injection into hind limb muscle. These experiments clearly showed evidence of retrograde spread of Lewy pathology through known neuroanatomically connected pathways in the motor system in a time-dependent manner (Fig. 7).

### Retrograde intraneuronal propagation of Lewy pathology

The direction of Lewy pathology propagation has been the subject of intensive investigation. Evidence for both anterograde [2, 42] and retrograde [9, 10, 28, 79] spread has been found in human disease tissue at autopsy, suggesting that both possibilities can occur. Modeling Lewy pathology in rodents has also suggested that spreading can occur in both the anterograde and retrograde direction [52], and several groups have suggested that retrograde spread may be the predominant mode [50, 59], especially initially [52]. However, a fundamental issue with cross-sectional analysis of pathology propagation, either in human disease or mouse model tissue, is the difficulty of extrapolating the time course of events for a particular cell from changes in group averages over time. The ability to use in vivo multiphoton imaging in our system allows us to perform longitudinal analyses of individual neurons in the mouse cortex during the entire time course of Lewy pathology formation and propagation in this new model system. In an analysis of > 700 cortical cells using this approach, our data clearly showed that the dominant mode of propagation starts with aggregation in axons and then spreads in a continuous and retrograde fashion to the soma. It was often possible to predict the future development of a somatic Lewy inclusion in a particular neuron several days before the somatic inclusion appeared because the axonal inclusion eventually approached its parent soma. From that point on somatic inclusion formation developed in a stereotyped fashion, with soluble alpha-synuclein clearing from the nucleus and then forming aggregates in the soma over the course of ~ 24 h (Additional file 12: Fig. S9). Importantly, in our analysis we never detected evidence of spread from the soma into the axon, suggesting that if this does occur in our model, it must occur at a very low rate. Certain previous work in human tissue analyzing the pattern of Lewy pathology propagation suggested that spread does occur primarily in a retrograde fashion [79–81], and our work is consistent with this hypothesis. This suggests that a specific species of alpha-synuclein, competent to seed the formation of Lewy pathology, is first taken up by neurons somewhere along the axon or in the presynaptic terminal. This “seed,” presumably a small, fibrillar form of alpha-synuclein, then recruits endogenous alpha-synuclein in the axonal compartment to begin the process of inclusion formation, which then spreads retrogradely back to the soma. Identifying the exact nature of this seed, how it first enters into axons, and how it then is capable of recruiting endogenous alpha-synuclein to form neuritic Lewy pathology will be important for future studies. The A53T SynGFP mouse model we have created will be useful in these endeavors.

### Peripheral to central nervous system propagation

Braak and colleagues were the first to suggest that Lewy pathology may form in the peripheral nervous system and then propagate centrally [9, 10] based on the analysis of human pathological tissue at various stages of disease. In these studies, the enteric nervous system and olfactory system were proposed as routes of entry into the CNS [28]. Recent work using PFFs to model Lewy pathology spread from either the peripheral olfactory or enteric nervous systems has shown conflicting results, with some groups unable to demonstrate robust and progressive peripheral-to-central transmission starting either in the peripheral olfactory epithelium [51] or enteric nervous system [31, 49, 82], and another showing that under certain circumstances such transmission is possible from the enteric nervous system [34]. In contrast, peripheral transmission from muscle PFF injections into spinal cord and brainstem motor structures has been reported [63], and we have also reproduced this result. In addition, our A53T SynGFP model has the advantage that within 8 months after intramuscular PFF injection Lewy pathology had spread further into the motor system, including the motor cortex. We interpret these findings as strongly suggesting that cell-to-cell propagation in this system is primarily through trans-synaptic spread, and that other putative mechanisms, such as transport by circulating microglia [37, 64, 69] or movement in the extracellular space [15, 39, 55] are less likely. This is the most parsimonious explanation for how pathology can progressively spread from muscle to spinal cord, to motor brainstem, and to motor cortex over time. Our model may be useful in future studies that aim to understand the molecular and cell biological basis for trans-synaptic spread.

One particular question of interest in relation to the trans-synaptic spread and retrograde propagation of Lewy pathology that we detect in our A53T SynGFP model after alpha-synuclein PFF injection is whether other, non-PFF-based models of progressive alpha-synuclein aggregation exhibit similar mechanisms. Substantial work has been done characterizing progressive, potentially trans-synaptic, spread of alpha-synuclein aggregation after oral exposure of rodents to the neurotoxin BSSG [32, 33, 73] and formation of aggregates in dopamine neurons after administration of bacterial LPS with LPS-simulated autologous macrophages [67]. It will be important in future studies to determine whether these relevant, toxin-induced forms of parkinsonism use similar trans-synaptic and retrograde Lewy pathology propagation mechanisms. Combining the A53T SynGFP model with these toxin-based induction models could directly address these questions.

### Astrocyte alpha-synuclein inclusion pathology

Our model demonstrates that within days after injection of PFFs into cortex, neurons begin to form Lewy inclusions. Once a neuron has formed an inclusion, however, it degenerates within several days to weeks. This pattern is accelerated in time course, but in general is consistent with our previous work studying Lewy pathology formation and consequences in vivo in a WT SynGFP mouse model [54]. After this first phase of primarily neuronal inclusion formation, the A53T SynGFP mouse developed alpha-synuclein aggregates in astrocytes and to a lesser extent, microglia. The presence of alpha-synuclein inclusions in astrocytes and microglia is not new. Several groups have shown that glial inclusions are present in human PD and DLB tissue, including subjects with SNCA gene triplication [11, 14, 25, 48, 78, 85] and following intracerebral injection of PFFs in the hippocampus [62] and striatum [71] and in a variety of transgenic mouse models [77]. Interestingly, the astrocytes bearing inclusions survive much longer than neurons bearing inclusions in A53T SynGFP mice. This astrocyte pathology may represent the seeding of inclusion formation within astrocytes, as occurs in neurons, or may be the result of phagocytic activity by these cell types that are clearing debris from stressed or dying inclusion-bearing neurons [1, 38, 41, 87]. Our current studies cannot distinguish between these two possibilities, but the delayed time course of astrocyte inclusion formation, the fact that few astrocytes express transgenic alpha-synuclein before PFF injection, and previous work by others showing in vitro and ex vivo neuron to astrocyte transfer of alpha-synuclein [43], suggest to us that the phagocytic functions of these cells may contribute to astrocyte inclusions. Therefore, it is possible that there are different mechanisms responsible for inclusion formation and pathological spread depending on the cell type involved and time since the initial seeding event. It is necessary to more thoroughly explore the role of glial cells, in particular astrocytes, in this process [72]. The A53T SynGFP model may be useful for future studies to understand the mechanisms of neuronal cell death and the contribution glial cells play in either limiting or exacerbating Lewy pathology.

In summary, we have developed a novel A53T SynGFP model that is particularly useful for studies of alpha-synuclein aggregation and propagation in the mammalian nervous system. Initial characterization of this model has provided clues to long-standing questions in the field, including direct evidence for retrograde axonal spread of Lewy pathology within neurons, and progressive trans-synaptic spread between neurons that can start in the peripheral nervous system and propagate into the CNS. This makes the A53T SynGFP model a useful new tool to expand our understanding of the mechanisms involved in aggregation and propagation of alpha-synuclein and to test how disease-specific mechanisms can be targeted therapeutically in the future.

## Supplementary information

**Additional file 1: Table S1.** Key Resources Table. All key resources are listed in the table above. Research Resource Identifiers (RRIDs) were available for all antibodies, along with the specialty assays, and software used. In addition, key reagents, dyes and the A53T SynGFP mouse line characterized in this manuscript are listed.

**Additional file 2: Table S2.** A53T SynGFP mice show both intra and inter-animal variability in the number and density of inclusions formed. A total of six A53T SynGFP mice were tracked in this study (1-6). The number and location of the specific areas visualized differed from mouse to mouse and was based on the initial injection site and the clarity of the individual cranial windows. Total number of areas imaged, total number of inclusions per animal, average number of inclusions per area (mean (SEM)) and average density (inclusions/mm3) (mean (SEM)) are shown for each mouse in rows 2-7. Group data is shown in row 8.

**Additional file 3: Table S3.** Mean and SEM group data following Intramuscular PFF injection. A total of 8 A53T SynGFP mice were included in this analysis, with 3-4 animals per group. Regions of interest (ROIs) were determined for 12 groups consisting of 3 brain areas (cortex, midbrain, pons) in in two conditions (motor and control), at 2 timepoints (4 and 8 months4- and 8-months post-injection (mpi)). The number and location of the specific ROIs differed from mouse to mouse based on subtle variations in serial sectioning. The number of regions of interest (ROIs) analyzed, the mean, and SEM of the density of inclusions in each ROI (mm2) are included in columns 2-4.

**Additional file 4: Figure S1.** Electron Micrographs and CLEM images show that A53T SynGFP localizes to presynaptic terminals in the striatum and cortex. **a** DAB/p-129 alpha-synuclein from the striatum of a SynGFP mouse. DAB labeling is present in presynaptic terminals surrounding synaptic vesicle structures. Scale bar 500 nm. **b** Inset from Fig. S1a demonstrating an example of DAB/p-129 alpha-synuclein labeled vesicles in a nerve terminal (NT) making an asymmetrical synaptic contact (arrow) onto an underlying dendritic spine (SP). Scale bar 500 nm. **c** Electron Microscopy (EM) image from CLEM processed tissue from the cortex of a SynGFP mouse. Scale bar 500 nm. **d** Inset from Fig. S1c showing two nerve terminals (NT) making asymmetrical synaptic contacts (arrows) onto a dendrite (DEND). Scale bar 500 nm. **e** The same EM image as Fig. S1c with an overlay of the fluorescent SynGFP signal captured from the same location using MAPS software creating a Correlated Light and Electron Microscopy (CLEM) image. SynGFP image localizes to vesicles in presynaptic terminals. Scale bar 500 nm. **f** Inset from Fig. S1e depicting a CLEM image of the same location shown in Fig. S1d with co-localization of the fluorescent SynGFP signal with vesicles in two nerve terminals (NT) making asymmetrical synaptic contacts (arrows) onto a dendrite (DEND). Scale bar 500 nm.

**Additional file 5: Figure S2.** PFF injection into Thy1-GFP transgenic mice does not induce GFP-positive Lewy pathology. **a** Top: PFF injection into A53T SynGFP Tg mice induces robust GFP-positive Lewy pathology 40 days post-injection that colocalizes well with the established Lewy marker pSyn. Bottom: PFF injection into GFP-only Tg mice induces less robust pSyn-positive Lewy pathololgy 4 months post-injection that does not colocalize well with GFP, demonstrating that it is composed of endogenous mouse alpha-synuclein. Scale bar 50 µm. **b** Left: A single A53T SynGFP Lewy inclusion shown at different planes in the Z-axis. Middle: Inclusion from a GFP-only animal shown in similar fashion. Right: Group data of Lewy pathology in A53T SynGFP Tg and GFP-only Tg mice, limited to neurons that express the respective transgene, shows a high level of colocalization between GFP fluorescence and pSyn only in A53T Syn-GFP animals (Pearson’s coefficient: A53T SynGFP-pSyn 0.81 ± 0.05%, GFP-pSyn: 0.25 ± 0.06; unpaired *t* test p < 0.0001; N = 3-5 cells/3 animals per group), demonstrating that even within neurons that have endogenous mouse alpha-synuclein inclusions and that express the GFP-only transgene, there is no incorporation of GFP into the inclusion. Scale bar 5 µm.

**Additional file 6: Figure S3.** Cortical Lewy pathology induced by PFF injection into A53T SynGFP mice is associated with cell death. **a** Left: WT mouse cortex at postnatal day 10, when developmental programmed cell death is known to occur, shows TUNEL positive cells with no aggregated pSyn Lewy pathology (positive control). Middle: A53T SynGFP cortex 40 days post-PFF injection shows TUNEL positive cells bearing somatic pSyn Lewy inclusions. Inset highlights example shown in yellow rectangle at higher magnification. Right: Uninjected A53T SynGFP cortex shows no TUNEL positive cells and no somatic Lewy pathology. Several nuclei are enriched with pSyn staining. Scale bar 50 µm. **b** Group data showing percent of nuclei that are TUNEL positive in each group (P10-11: 0.87 ± 0.41%, A53T SynGFP + PFF: 0.63 ± 0.39%, A53T SynGFP: 0.0 ± 0.0%; one-way ANOVA (F(2, 12) = 7.035, p = 0.0095), post hoc Tukey tests: P10-11 vs. A53T SynGFP + PFF p = 0.5153, P10-11 vs. A53T SynGFP p = 0.0096, A53T SynGFP + PFF vs. A53T SynGFP p = 0.0319; N = 4-7 ROIs/2-3 animals per group).

**Additional file 7: Figure S4.** PFF but not monomeric alpha-synuclein injection into mouse brain induces Lewy pathology. **a** Monomer or PFF striatal injections were done in A53T SynGFP animals at 5-8 months-old, with sacrifice 9 months later (14-17 months old). Brain sections were processed for DAB immunohistochemistry, labeling pSyn-positive Lewy pathology. Top row: Monomer injections showed background pSyn labeling in striatum, subcortical white matter, and motor cortex, demonstrating no detectable Lewy pathology due to spontaneous formation or induced by injection of monomeric alpha-synuclein. Bottom row: In contrast, PFF injections demonstrated robust formation of pSyn-positive dystrophic neurites and cells, indicative of Lewy pathology, in the striatum, subcortical white matter, and motor cortex. **b** Group data shows a significant increase in pSyn intensity in each indicated brain region in PFF versus monomer injections (two-way ANOVA (interaction F(2, 48) = 11.39), (brain region F(2, 48) = 19.99), (monomer vs. PFF F(1, 48) = 295.0), p < 0.0001 for all three; Sidak’s multiple comparisons test, monomer vs. PFF: striatum, subcortical white matter, and cortex p < 0.0001 for all three; N = 9 ROIs/3 animals per group).

**Additional file 8: Figure S5.** A53T SynGFP aggregates appear first in presumed axons (Video). **a** Video showing in vivo multiphoton 3D projection of serial images of the same neuron at progressive time points after PFF injection. The orientation of the neuron in the video can be viewed at 0:00:03. At this timepoint the pial surface is at the top of the image and the axon can be seen pointing down towards the deeper cortical layers (still image shown in Fig. 5). A53T SynGFP aggregates (increased GFP intensity shown in green) form first in the axon and predict formation of a somatic inclusion in this neuron.

**Additional file 9: Figure S6.** A53T SynGFP inclusions are present in neuritic structures but do not co-localize with tested axonal or dendritic markers. **a** Maximum intensity projection of a neuritic inclusion and anti-neurofilament antibody stain (SMI312). Scale bar 5 µm. **b** The vast majority of neurites are not recognized by either the anti-neurofilament stain (SMI312), or the anti-MAP2 stain (MAP2), although significant increase in staining in some SMI312-positive axons was found compared to MAP2-positive dendrites (Chi-square (1) = 3.932, p = 0.0474, N = 110 neurites).

**Additional file 10: Figure S7.** Syn-mRuby PFFs transported along axons. Cartoon, adapted from Paxinos [58], depicting the location of Syn-mRuby PFFs (red objects) relative to injection site (arrow head) over 4 weeks. Insets: Fixed tissue fluorescent images of Syn-mRuby signal along apparent axons (arrows). Scale bar 20 µm.

**Additional file 11: Figure S8.** A53T SynGFP inclusions appear in TMEM119-positive cells at a similar percentage as Iba1-positive cells. **a** Example A53T SynGFP inclusion in a TMEM119-positive cell. The morphology of the TMEM119-positive inclusions was variable. However, a larger number of TMEM119-positive SynGFP inclusions did co-stained with pSyn, unlike the Iba1-positive SynGFP inclusions (Fig. 6c) Scale bar 10 µm. **b** Group data shows that the cell-type dependence of A53T SynGFP inclusions does not differ in Iba1-postive and TMEM119-positive cells (two-way ANOVA (interaction F(1,20) = 0.5451), (Early vs. Late F(1, 20) = 3.044), (cell type F(1,20) = 2.056), p = 0.4689, p = 0.0964, and p = 0.1670, respectively; N = 6 animals per timepoint). Early intervals post-injection equal less than 50 days post-injection (dpi). Late intervals post-injection equal greater than 50 dpi. Mean and SEM of percentage of inclusions from each slice analyzed from each animal are shown at each timepoint. Iba1 = blue and TMEM119 = black. **c** Example of a large SynGFP-positive aggregate being engulfed by the process of a TMEM119-positive microglia. These large SynGFP-positive aggregates were not classified as inclusions in our analysis because they were not associated with a single DAPI-positive nucleus and instead appeared to be free in the neuropil. Scale bar 10 µm.

**Additional file 12: Figure S9.** Somatic inclusions form quickly and developed in a rather stereotyped fashion. In vivo multiphoton image showing serial sections of the same neuron at different depths within a single stack at 2 consecutive days after PFF injection. A53T SynGFP aggregates form first as a small punctum in the likely axon and predicts formation of a somatic inclusion in this neuron. Small puncta (white arrow) increases intensity (day 8) as the homogenous fluorescent signal in the cell body (yellow arrow) clears and begins to form a mature somatic inclusion.
