# Supplementary material for: Trans-synaptic and retrograde axonal spread of Lewy pathology following pre-formed fibril injection in an in vivo A53T alpha-synuclein mouse model of synucleinopathy
Source: Acta Neuropathol Commun. 2020 Aug 28;8:150. doi: 10.1186/s40478-020-01026-0 (PMC7456087; doi:10.1186/s40478-020-01026-0)
Supplement: Supplementary file 1 — Additional file 1: Table S1. Key Resources Table. All key resources are listed in the table above. Research Resource Identifiers (RRIDs) were available for all antibodies, along with the specialty assays, and software used. In addition, key reagents, dyes and the A53T SynGFP mouse line characterized in this manuscript are listed. [file 40478_2020_1026_MOESM1_ESM.docx]

**KEY RESOURCES TABLE**

| REAGENT or RESOURCE | SOURCE | IDENTIFIER | RRID |
| --- | --- | --- | --- |
| Antibodies | | |  |
| Mouse anti-α-Synuclein Phospho (Ser129) (1:667, IHC; 1:500, EM) | Biolegend | Cat# 825701 | AB_2564891 |
| Rabbit anti-alpha Synuclein (phospho S129) [EP1536Y] (1:500/mouse;1:10,000/human, IHC) | Abcam | Cat# ab51253 | AB_869973 |
| Mouse anti-aSYN (Syn303)/oxidized (1:10,000, IHC) | Biolegend | Cat# 824301 | AB_2564879 |
| Mouse anti-Nitrated a-Synuclein (Syn505)(1:10,000, IHC) | Thermo Fisher Scientific | Cat# 35-8300 | AB_2533225 |
| Rabbit Anti-Ubiquitin (1:200/mouse; 1:10,000/human, IHC) | Dako | Cat# Z5116 | AB_2622233 |
| Rabbit Anti-GFP (1:500, IHC) | Abcam | Cat# ab6556 | AB_305564 |
| Rabbit Anti-cleaved Caspase-3 (1:500, IHC) | Cell Signaling | Cat # 9662 | AB_331439 |
| X-34 dye | Klunk Lab | [1] |  |
| Rabbit NeuN Polyclonal (1:500, IHC) | ABclonal | Cat# A3051 | AB_2764855 |
| Mouse Glial Fibrillary Acidic Protein (GFAP) (1:300, IHC) | Spring Bioscience | Cat# E16510 | AB_1661180 |
| Rabbit Anti-Iba1 antibody [EPR16588] (1:500, IHC) | Abcam | Cat# ab178846 | AB_2636859 |
| Rabbit Anti-TMEM119 antibody (1:500, IHC) | Synaptic Systems | Cat# 400 003 | AB_2725756 |
| biotinylated goat anti-mouse (1:100; EM) | Jackson ImmunoResearch | Cat# 115-065-003 | AB_2338557 |
| goat anti-rabbit Alexa 647 (1:1000, IHC) | Molecular Probes/Invitrogen | cat# MPA21245 | AB_2535862 |
| goat anti-mouse Alexa 647 (1:1000, IHC) | Molecular Probes/Invitrogen | cat# MPA21236 | AB_141725 |
| goat anti-rabbit Alexa 555 (1:1000, IHC) | Abcam | cat# ab150078 | AB_2722519 |
| goat anti-mouse Alexa 555 (1:1000, IHC) | Abcam | cat# ab150114 | AB_2687594 |
| Chemicals, Peptides, and Recombinant Proteins | | |  |
| LR White Resin | Electron Microscopy Science | cat# 14382 |  |
| Critical Commercial Assays | | |  |
| Pelco Biowave Microwave Pro | Ted Pella | cat# 36700 | SCR_018608 |
| In situ Cell Death Detection Kit, TMR red | Roche | Cat# 12156792910 |  |
| Experimental Models: Organisms/Strains | | |  |
| Mouse: A53T SynGFP | OHSU Transgenic Mouse Model Core |  |  |
| Software/Algorithms | | |  |
| ZEN Blue | Zeiss |  | SCR_013672 |
| MAPS 2.0 | FEI |  | SCR_018738 |
| ImageJ/FIJI | Reference [58] | https://fiji.sc/ | SCR_002285 |
| Imaris | Bitplane |  | SCR_002798 |
| Prism 8 | Graphpad Software, Inc. |  | SCR_002798 |

**Table S1** Key Resources Table. All key resources are listed in the table above. Research Resource Identifiers (RRIDs) were available for all antibodies, along with the specialty assays, and software used. In addition, key reagents, dyes and the A53T SynGFP mouse line characterized in this manuscript are listed.

1. Styren SD, Hamilton RL, Styren GC, Klunk WE (2000) X-34, a fluorescent derivative of Congo red: A novel histochemical stain for Alzheimer’s disease pathology. J Histochem Cytochem 48:1223–1232. doi: 10.1177/002215540004800906
