# Supplementary material for: Trans-synaptic and retrograde axonal spread of Lewy pathology following pre-formed fibril injection in an in vivo A53T alpha-synuclein mouse model of synucleinopathy
Source: Acta Neuropathol Commun. 2020 Aug 28;8:150. doi: 10.1186/s40478-020-01026-0 (PMC7456087; doi:10.1186/s40478-020-01026-0)
Supplement: Supplementary file 2 — Additional file 2: Table S2. A53T SynGFP mice show both intra and inter-animal variability in the number and density of inclusions formed. A total of six A53T SynGFP mice were tracked in this study (1-6). The number and location of the specific areas visualized differed from mouse to mouse and was based on the initial injection site and the clarity of the individual cranial windows. Total number of areas imaged, total number of inclusions per animal, average number of inclusions per area (mean (SEM)) and average density (inclusions/mm3) (mean (SEM)) are shown for each mouse in rows 2-7. Group data is shown in row 8. [file 40478_2020_1026_MOESM2_ESM.docx]

| **Animal Number (Sex)** | **Areas Imaged** | | | **Total Inclusions**  **Per Animal** | | | **Avg. # of Inclusions**  **Per Area** | | | **Avg. Density (inclusions/mm^3^)** | | | |
| --- | --- | --- | --- | --- | --- | --- | --- | --- | --- | --- | --- | --- | --- |
|  | *Total* | *Ipsi* | *Contra* | *Total* | *Ipsi* | *Contra* | *Total* | *Ipsi* | *Contra* | *Total* | *Ipsi* | *Contra* |  |
| *1 (F)* | 6 | 0 | 6 | 73 | 0 | 73 | 12.17  (2.96) | 0.00 | 12.17  (2.96) | 1569.84  (362.08) | 0.00 | 1569.84 (362.08) |  |
| *2 (F)* | 5 | 3 | 2 | 832 | 719 | 113 | 166.5  (66.63) | 239.67  (89.08) | 56.50  (21.50) | 14813.10  (5373.98) | 20683.60  (7249.42) | 6007.36  (1377.97) |  |
| *3 (F)* | 7 | 4 | 3 | 498 | 474 | 24 | 71.14  (22.99) | 118.50  (16.39) | 8.00  (1.16) | 7278.03  (2298.30) | 11828.51  (1538.92) | 1210.73  (167.36) |  |
| *4 (M)* | 6 | 3 | 3 | 482 | 205 | 277 | 80.33  (11.16) | 68.33  (17.32) | 92.33  (13.35) | 9827.16  (1432.00) | 7601.63  (1409.20) | 12052.69 (1820.52) |  |
| *5 (M)* | 7 | 4 | 3 | 144 | 64 | 80 | 20.57  (6.286) | 16.00  (6.78) | 26.67  (12.33) | 1936.17  (464.09) | 1536.43  (543.53) | 2469.16 (817.47) |  |
| *6 (F)* | 6 | 1 | 5 | 209 | 39 | 170 | 34.83  (7.195) | 39.00  (0.00) | 34.00  (8.75) | 3489.80  (739.74) | 3412.92  (0.00) | 3505.17 (905.80) |  |
| *Group Data* | 37 | 15 | 22 | 2238 | 1501 | 737 | 373  (116.6) | 300.2  (130.1) | 122.8  (36.59) | 6808.64  (2315.56) | 9190.21  (3531.10) | 4449.42  (1677.59) |  |

**Table S2** A53T SynGFP mice show both intra and inter-animal variability in the number and density of inclusions formed. A total of six A53T SynGFP mice were tracked in this study (1-6). The number and location of the specific areas visualized differed from mouse to mouse and was based on the initial injection site and the clarity of the individual cranial windows. Total number of areas imaged, total number of inclusions per animal, average number of inclusions per area (mean (SEM)) and average density (inclusions/mm3) (mean (SEM)) are shown for each mouse in rows 2-7. Group data is shown in row 8.

*** Inclusion formation results were not dependent on age.
Total inclusions per animal: simple linear regression (F(1,4)=3.814, p=0.1225), with an R^2^  of 0.4881
Avg. Density: simple linear regression (F(1,4)=2.950, p=0.610), with an R^2^ of 0.4345
