# Supplementary material for: Trans-synaptic and retrograde axonal spread of Lewy pathology following pre-formed fibril injection in an in vivo A53T alpha-synuclein mouse model of synucleinopathy
Source: Acta Neuropathol Commun. 2020 Aug 28;8:150. doi: 10.1186/s40478-020-01026-0 (PMC7456087; doi:10.1186/s40478-020-01026-0)
Supplement: Supplementary file 3 — Additional file 3: Table S3. Mean and SEM group data following Intramuscular PFF injection. A total of 8 A53T SynGFP mice were included in this analysis, with 3-4 animals per group. Regions of interest (ROIs) were determined for 12 groups consisting of 3 brain areas (cortex, midbrain, pons) in in two conditions (motor and control), at 2 timepoints (4 and 8 months4- and 8-months post-injection (mpi)). The number and location of the specific ROIs differed from mouse to mouse based on subtle variations in serial sectioning. The number of regions of interest (ROIs) analyzed, the mean, and SEM of the density of inclusions in each ROI (mm2) are included in columns 2-4. [file 40478_2020_1026_MOESM3_ESM.docx]

| **Group** | **# of ROIs Analyzed** | **Mean (inclusion #/mm^2^)** | **SEM** |
| --- | --- | --- | --- |
| Control ROI Cortex 4mpi | 9 | 1.000 | 1.118 |
| Motor ROI Cortex 4mpi | 18 | 4.111 | 1.114 |
| Control ROI Midbrain 4mpi | 20 | 0.8000 | 0.2128 |
| Motor ROI Midbrain 4mpi | 20 | 18.55 | 2.858 |
| Control ROI Pons 4mpi | 6 | 0.1667 | 0.1667 |
| Motor ROI Pons 4mpi | 10 | 38.00 | 6.254 |
| Control ROI Cortex 8mpi | 10 | 1.600 | 0.4000 |
| Motor ROI Cortex 8mpi | 12 | 45.33 | 5.578 |
| Control ROI Midbrain 8mpi | 15 | 1.933 | 0.7202 |
| Motor ROI Midbrain 8mpi | 16 | 91.31 | 11.02 |
| Control ROI Pons 8mpi | 6 | 0.5000 | 0.2236 |
| Motor ROI Pons 8mpi | 8 | 140.5 | 21.43 |

**Table S3** Mean and SEM group data following Intramuscular PFF injection. A total of 8 A53T SynGFP mice were included in this analysis, with 3-4 animals per group. Regions of interest (ROIs) were determined for 12 groups consisting of 3 brain areas (cortex, midbrain, pons) in in two conditions (motor and control), at 2 timepoints (4 and 8 months4- and 8-months post-injection (mpi)). The number and location of the specific ROIs differed from mouse to mouse based on subtle variations in serial sectioning. The number of regions of interest (ROIs) analyzed, the mean, and SEM of the density of inclusions in each ROI (mm2) are included in columns 2-4.
