# Supplementary figures and images for: Trans-synaptic and retrograde axonal spread of Lewy pathology following pre-formed fibril injection in an in vivo A53T alpha-synuclein mouse model of synucleinopathy
Source: Acta Neuropathol Commun. 2020 Aug 28;8:150. doi: 10.1186/s40478-020-01026-0 (PMC7456087; doi:10.1186/s40478-020-01026-0)

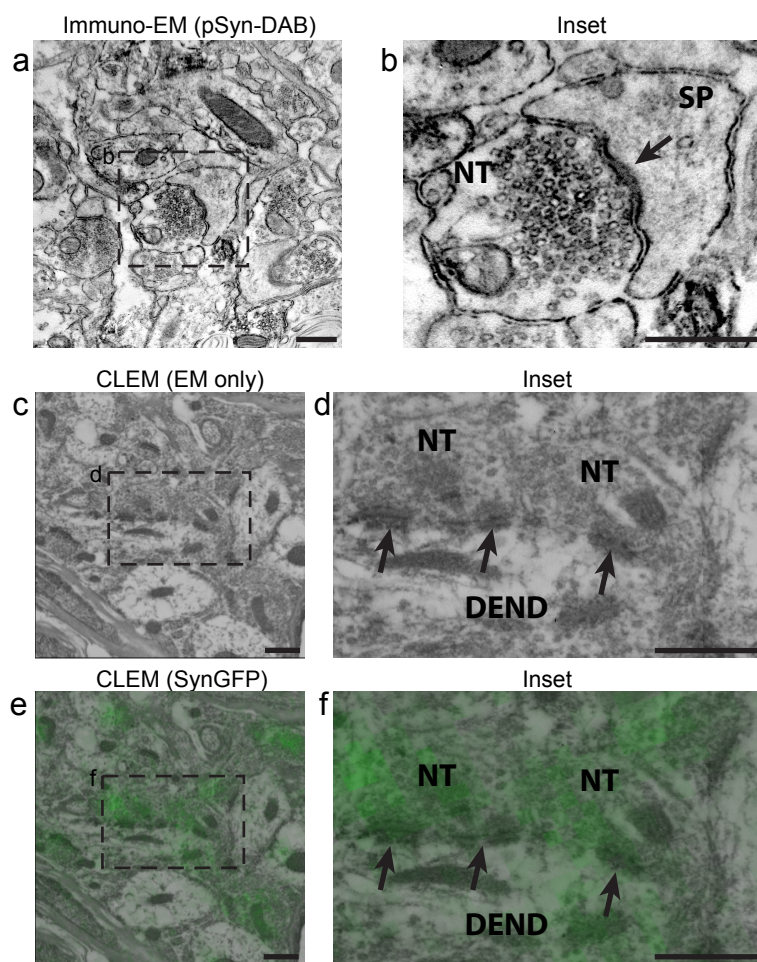

Supplement: Supplementary file 4 — Additional file 4: Figure S1. Electron Micrographs and CLEM images show that A53T SynGFP localizes to presynaptic terminals in the striatum and cortex. a DAB/p-129 alpha-synuclein from the striatum of a SynGFP mouse. DAB labeling is present in presynaptic terminals surrounding synaptic vesicle structures. Scale bar 500 nm. b Inset from Fig. S1a demonstrating an example of DAB/p-129 alpha-synuclein labeled vesicles in a nerve terminal (NT) making an asymmetrical synaptic contact (arrow) onto an underlying dendritic spine (SP). Scale bar 500 nm. c Electron Microscopy (EM) image from CLEM processed tissue from the cortex of a SynGFP mouse. Scale bar 500 nm. d Inset from Fig. S1c showing two nerve terminals (NT) making asymmetrical synaptic contacts (arrows) onto a dendrite (DEND). Scale bar 500 nm. e The same EM image as Fig. S1c with an overlay of the fluorescent SynGFP signal captured from the same location using MAPS software creating a Correlated Light and Electron Microscopy (CLEM) image. SynGFP image localizes to vesicles in presynaptic terminals. Scale bar 500 nm. f Inset from Fig. S1e depicting a CLEM image of the same location shown in Fig. S1d with co-localization of the fluorescent SynGFP signal with vesicles in two nerve terminals (NT) making asymmetrical synaptic contacts (arrows) onto a dendrite (DEND). Scale bar 500 nm. [file 40478_2020_1026_MOESM4_ESM.pdf]

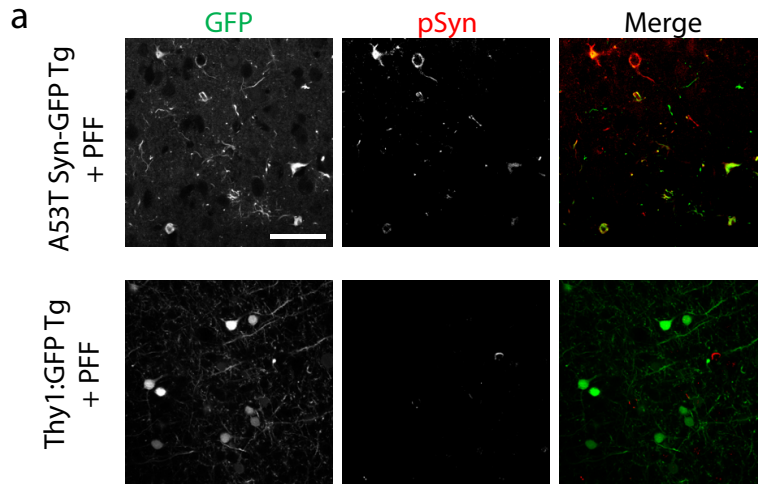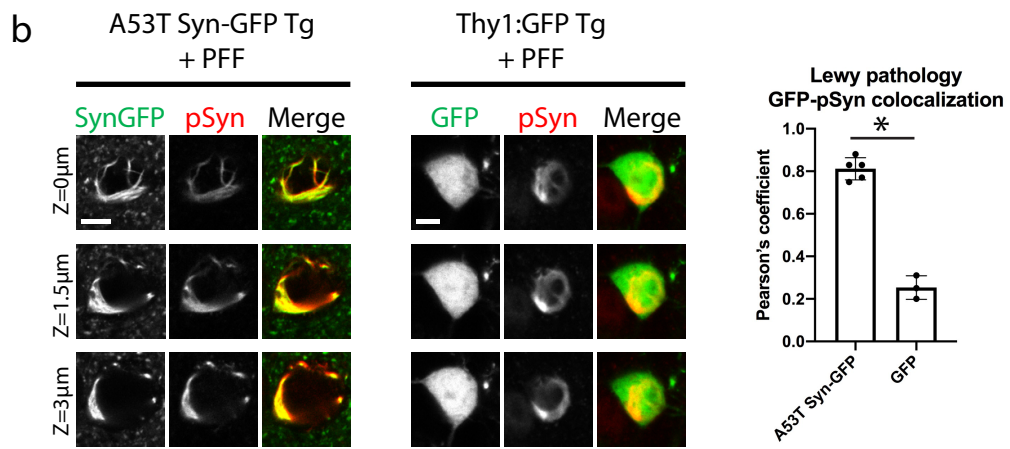

Supplement: Supplementary file 5 — Additional file 5: Figure S2. PFF injection into Thy1-GFP transgenic mice does not induce GFP-positive Lewy pathology. a Top: PFF injection into A53T SynGFP Tg mice induces robust GFP-positive Lewy pathology 40 days post-injection that colocalizes well with the established Lewy marker pSyn. Bottom: PFF injection into GFP-only Tg mice induces less robust pSyn-positive Lewy pathololgy 4 months post-injection that does not colocalize well with GFP, demonstrating that it is composed of endogenous mouse alpha-synuclein. Scale bar 50 µm. b Left: A single A53T SynGFP Lewy inclusion shown at different planes in the Z-axis. Middle: Inclusion from a GFP-only animal shown in similar fashion. Right: Group data of Lewy pathology in A53T SynGFP Tg and GFP-only Tg mice, limited to neurons that express the respective transgene, shows a high level of colocalization between GFP fluorescence and pSyn only in A53T Syn-GFP animals (Pearson’s coefficient: A53T SynGFP-pSyn 0.81 ± 0.05%, GFP-pSyn: 0.25 ± 0.06; unpaired t test p < 0.0001; N = 3-5 cells/3 animals per group), demonstrating that even within neurons that have endogenous mouse alpha-synuclein inclusions and that express the GFP-only transgene, there is no incorporation of GFP into the inclusion. Scale bar 5 µm. [file 40478_2020_1026_MOESM5_ESM.pdf]

**a**

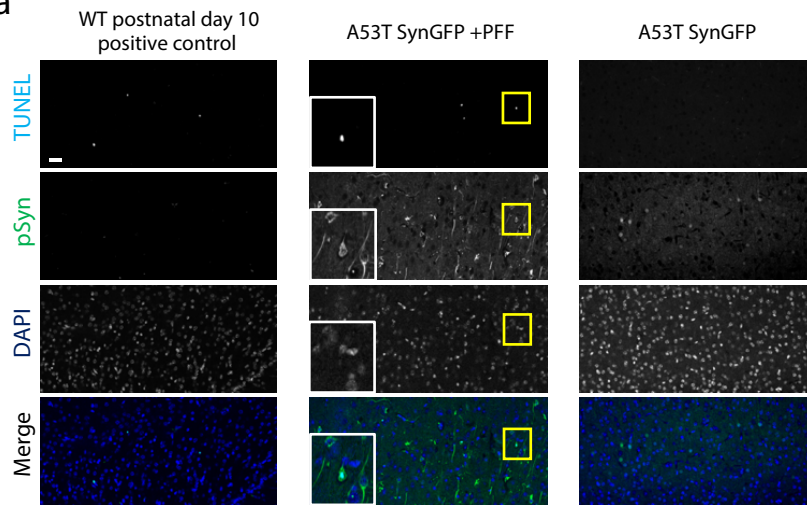

**b**

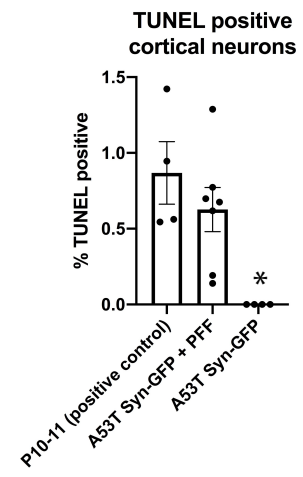

Supplement: Supplementary file 6 — Additional file 6: Figure S3. Cortical Lewy pathology induced by PFF injection into A53T SynGFP mice is associated with cell death. a Left: WT mouse cortex at postnatal day 10, when developmental programmed cell death is known to occur, shows TUNEL positive cells with no aggregated pSyn Lewy pathology (positive control). Middle: A53T SynGFP cortex 40 days post-PFF injection shows TUNEL positive cells bearing somatic pSyn Lewy inclusions. Inset highlights example shown in yellow rectangle at higher magnification. Right: Uninjected A53T SynGFP cortex shows no TUNEL positive cells and no somatic Lewy pathology. Several nuclei are enriched with pSyn staining. Scale bar 50 µm. b Group data showing percent of nuclei that are TUNEL positive in each group (P10-11: 0.87 ± 0.41%, A53T SynGFP + PFF: 0.63 ± 0.39%, A53T SynGFP: 0.0 ± 0.0%; one-way ANOVA (F(2, 12) = 7.035, p = 0.0095), post hoc Tukey tests: P10-11 vs. A53T SynGFP + PFF p = 0.5153, P10-11 vs. A53T SynGFP p = 0.0096, A53T SynGFP + PFF vs. A53T SynGFP p = 0.0319; N = 4-7 ROIs/2-3 animals per group). [file 40478_2020_1026_MOESM6_ESM.pdf]

a

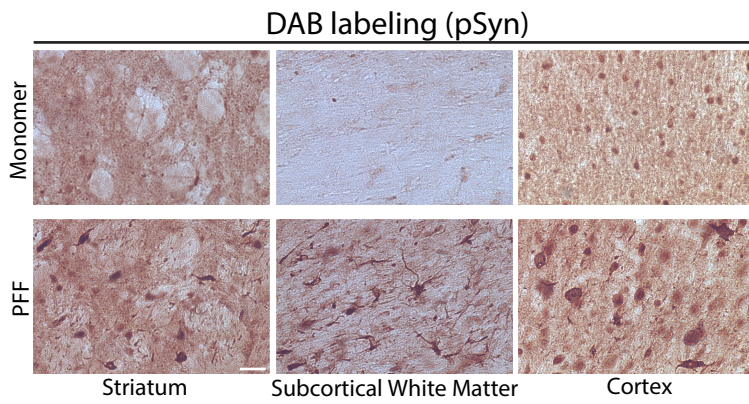

b

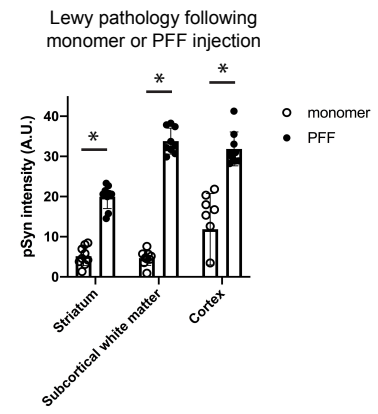

Supplement: Supplementary file 7 — Additional file 7: Figure S4. PFF but not monomeric alpha-synuclein injection into mouse brain induces Lewy pathology. a Monomer or PFF striatal injections were done in A53T SynGFP animals at 5-8 months-old, with sacrifice 9 months later (14-17 months old). Brain sections were processed for DAB immunohistochemistry, labeling pSyn-positive Lewy pathology. Top row: Monomer injections showed background pSyn labeling in striatum, subcortical white matter, and motor cortex, demonstrating no detectable Lewy pathology due to spontaneous formation or induced by injection of monomeric alpha-synuclein. Bottom row: In contrast, PFF injections demonstrated robust formation of pSyn-positive dystrophic neurites and cells, indicative of Lewy pathology, in the striatum, subcortical white matter, and motor cortex. b Group data shows a significant increase in pSyn intensity in each indicated brain region in PFF versus monomer injections (two-way ANOVA (interaction F(2, 48) = 11.39), (brain region F(2, 48) = 19.99), (monomer vs. PFF F(1, 48) = 295.0), p < 0.0001 for all three; Sidak’s multiple comparisons test, monomer vs. PFF: striatum, subcortical white matter, and cortex p < 0.0001 for all three; N = 9 ROIs/3 animals per group). [file 40478_2020_1026_MOESM7_ESM.pdf]

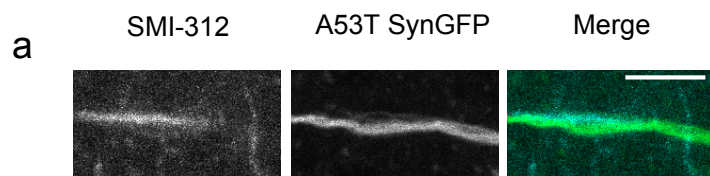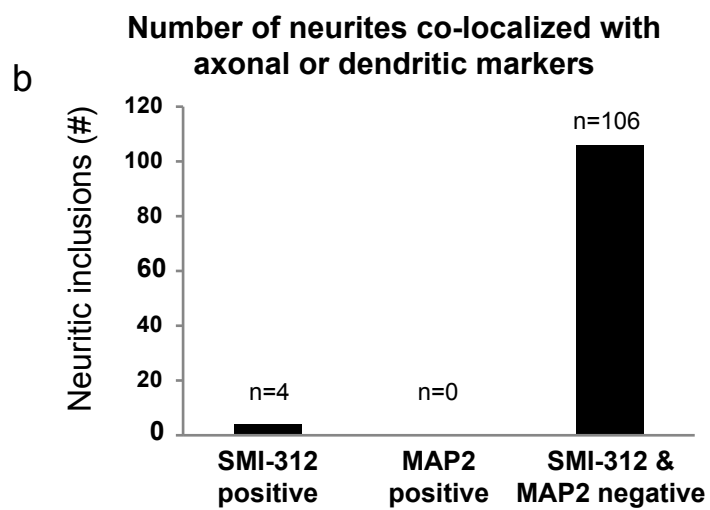

Supplement: Supplementary file 9 — Additional file 9: Figure S6. A53T SynGFP inclusions are present in neuritic structures but do not co-localize with tested axonal or dendritic markers. a Maximum intensity projection of a neuritic inclusion and anti-neurofilament antibody stain (SMI312). Scale bar 5 µm. b The vast majority of neurites are not recognized by either the anti-neurofilament stain (SMI312), or the anti-MAP2 stain (MAP2), although significant increase in staining in some SMI312-positive axons was found compared to MAP2-positive dendrites (Chi-square (1) = 3.932, p = 0.0474, N = 110 neurites). [file 40478_2020_1026_MOESM9_ESM.pdf]

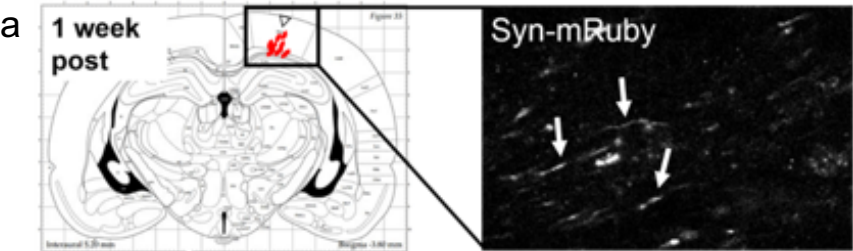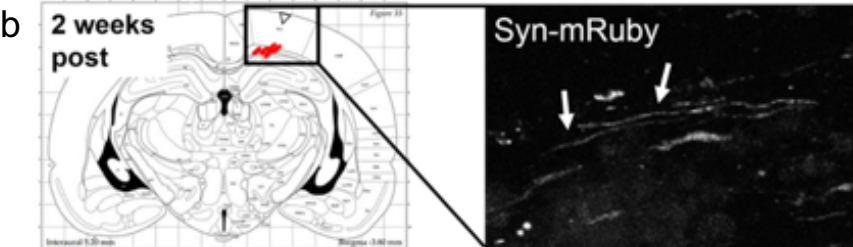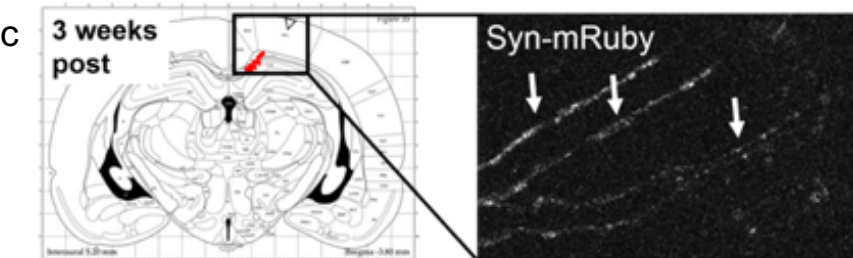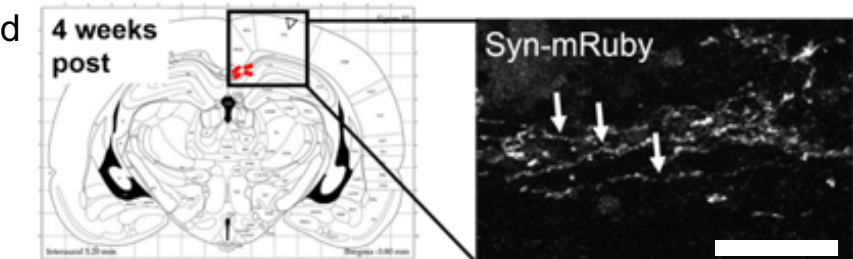

Supplement: Supplementary file 10 — Additional file 10: Figure S7. Syn-mRuby PFFs transported along axons. Cartoon, adapted from Paxinos [58], depicting the location of Syn-mRuby PFFs (red objects) relative to injection site (arrow head) over 4 weeks. Insets: Fixed tissue fluorescent images of Syn-mRuby signal along apparent axons (arrows). Scale bar 20 µm. [file 40478_2020_1026_MOESM10_ESM.pdf]

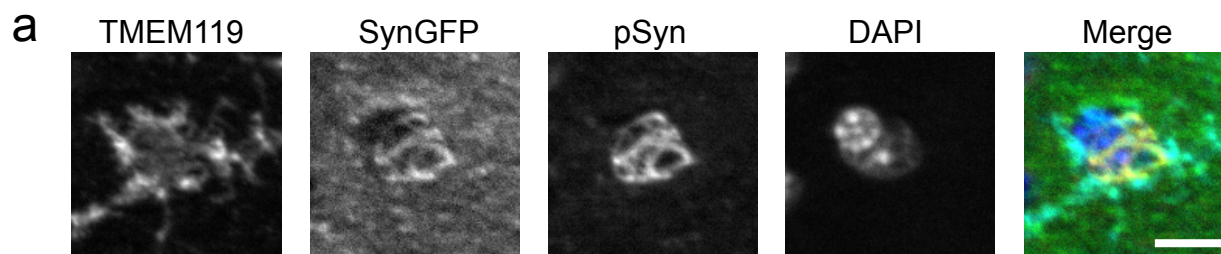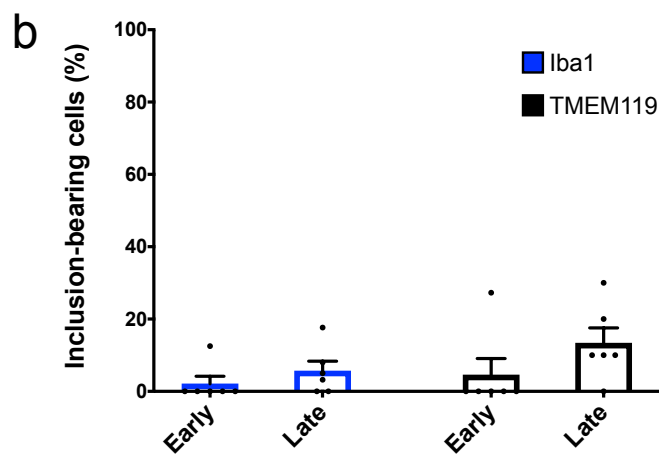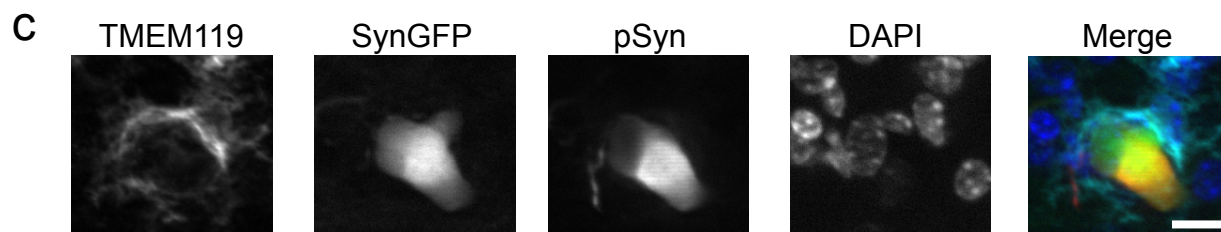

Supplement: Supplementary file 11 — Additional file 11: Figure S8. A53T SynGFP inclusions appear in TMEM119-positive cells at a similar percentage as Iba1-positive cells. a Example A53T SynGFP inclusion in a TMEM119-positive cell. The morphology of the TMEM119-positive inclusions was variable. However, a larger number of TMEM119-positive SynGFP inclusions did co-stained with pSyn, unlike the Iba1-positive SynGFP inclusions (Fig. 6c) Scale bar 10 µm. b Group data shows that the cell-type dependence of A53T SynGFP inclusions does not differ in Iba1-postive and TMEM119-positive cells (two-way ANOVA (interaction F(1,20) = 0.5451), (Early vs. Late F(1, 20) = 3.044), (cell type F(1,20) = 2.056), p = 0.4689, p = 0.0964, and p = 0.1670, respectively; N = 6 animals per timepoint). Early intervals post-injection equal less than 50 days post-injection (dpi). Late intervals post-injection equal greater than 50 dpi. Mean and SEM of percentage of inclusions from each slice analyzed from each animal are shown at each timepoint. Iba1 = blue and TMEM119 = black. c Example of a large SynGFP-positive aggregate being engulfed by the process of a TMEM119-positive microglia. These large SynGFP-positive aggregates were not classified as inclusions in our analysis because they were not associated with a single DAPI-positive nucleus and instead appeared to be free in the neuropil. Scale bar 10 µm. [file 40478_2020_1026_MOESM11_ESM.pdf]

Deeper Cortical Layers ← Distal/Proximal Axis → Pial Surface

Day 7

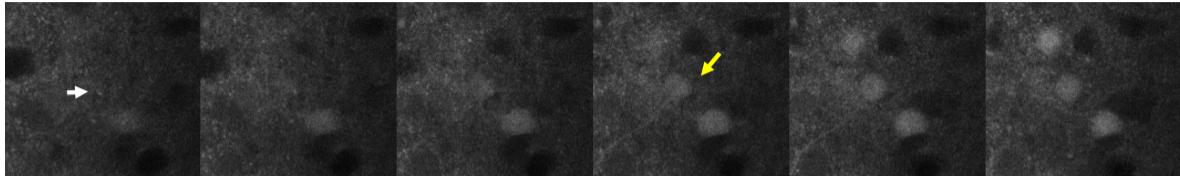

Day 8

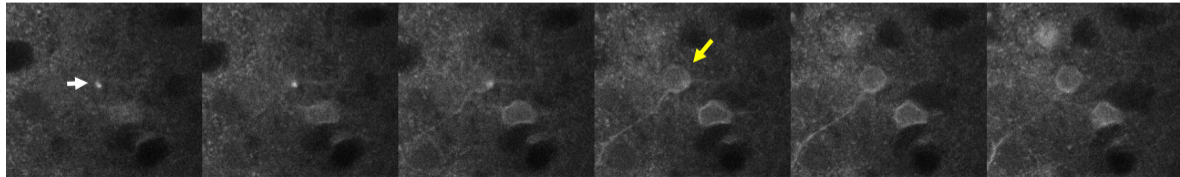

Supplement: Supplementary file 12 — Additional file 12: Figure S9. Somatic inclusions form quickly and developed in a rather stereotyped fashion. In vivo multiphoton image showing serial sections of the same neuron at different depths within a single stack at 2 consecutive days after PFF injection. A53T SynGFP aggregates form first as a small punctum in the likely axon and predicts formation of a somatic inclusion in this neuron. Small puncta (white arrow) increases intensity (day 8) as the homogenous fluorescent signal in the cell body (yellow arrow) clears and begins to form a mature somatic inclusion. [file 40478_2020_1026_MOESM12_ESM.pdf]
